# Predictors of In-Hospital Mortality after Thrombectomy in Anterior Circulation Large Vessel Occlusion: A Retrospective, Machine Learning Study

**DOI:** 10.3390/diagnostics14141531

**Published:** 2024-07-16

**Authors:** Ivan Petrović, Serena Broggi, Monika Killer-Oberpfalzer, Johannes A. R. Pfaff, Christoph J. Griessenauer, Isidora Milosavljević, Ana Balenović, Johannes S. Mutzenbach, Slaven Pikija

**Affiliations:** 1 Faculty of Medicine, University of Novi Sad, 21000 Novi Sad, Serbia; 014568@mf.uns.ac.rs (I.M.); 014631@mf.uns.ac.rs (A.B.); 2 Neurology and Stroke Unit, ASST dei Sette Laghi, 21100 Varese, Italy; serena.broggi@gmail.com; 3 Department of Neurology, University Hospital Salzburg, Christian Doppler Klinik, Paracelsus Medical University Salzburg, 5020 Salzburg, Austria; m.killer@salk.at (M.K.-O.); j.mutzenbach@salk.at (J.S.M.); 4 Institute of Neurointervention, Christian Doppler Klinik, Paracelsus Medical University Salzburg, 5020 Salzburg, Austria; 5 Department of Neuroradiology, University Hospital Salzburg, Christian Doppler Klinik, Paracelsus Medical University Salzburg, 5020 Salzburg, Austria; j.pfaff@salk.at; 6 Department of Neurosurgery, University Hospital Salzburg, Christian Doppler Klinik, Paracelsus Medical University, 5020 Salzburg, Austria; c.griessenauer@salk.at

**Keywords:** ischemic stroke, machine learning, mechanical thrombectomy, in-hospital mortality

## Abstract

Background: Despite the increased use of mechanical thrombectomy (MT) in recent years, there remains a lack of research on in-hospital mortality rates following the procedure, the primary factors influencing these rates, and the potential for predicting them. This study aimed to utilize interpretable machine learning (ML) to help clarify these uncertainties. Methods: This retrospective study involved patients with anterior circulation large vessel occlusion (LVO)-related ischemic stroke who underwent MT. The patient division was made into two groups: (I) the in-hospital death group, referred to as miserable outcome, and (II) the in-hospital survival group, or favorable outcome. Python 3.10.9 was utilized to develop the machine learning models, which consisted of two types based on input features: (I) the Pre-MT model, incorporating baseline features, and (II) the Post-MT model, which included both baseline and MT-related features. After a feature selection process, the models were trained, internally evaluated, and tested, after which interpretation frameworks were employed to clarify the decision-making processes. Results: This study included 602 patients with a median age of 76 years (interquartile range (IQR) 65–83), out of which 54% (*n* = 328) were female, and 22% (*n* = 133) had miserable outcomes. Selected baseline features were age, baseline National Institutes of Health Stroke Scale (NIHSS) value, neutrophil-to-lymphocyte ratio (NLR), international normalized ratio (INR), the type of the affected vessel (‘Vessel type’), peripheral arterial disease (PAD), baseline glycemia, and premorbid modified Rankin scale (pre-mRS). The highest odds ratio of 4.504 was observed with the presence of peripheral arterial disease (95% confidence interval (CI), 2.120–9.569). The Pre-MT model achieved an area under the curve (AUC) value of around 79% utilizing these features, and the interpretable framework discovered the baseline NIHSS value as the most influential factor. In the second data set, selected features were the same, excluding pre-mRS and including puncture-to-procedure-end time (PET) and onset-to-puncture time (OPT). The AUC value of the Post-MT model was around 84% with age being the highest-ranked feature. Conclusions: This study demonstrates the moderate to strong effectiveness of interpretable machine learning models in predicting in-hospital mortality following mechanical thrombectomy for ischemic stroke, with AUCs of 0.792 for the Pre-MT model and 0.837 for the Post-MT model. Key predictors included patient age, baseline NIHSS, NLR, INR, occluded vessel type, PAD, baseline glycemia, pre-mRS, PET, and OPT. These findings provide valuable insights into risk factors and could improve post-procedural patient management.

## 1. Introduction

Despite major advances in emergent treatment, stroke is still a devastating disease identified as one of the leading causes of death and disability on a global level [1,2,3,4,5,6,7,8]. Acute ischemic stroke (AIS), caused by the culprit cessation of brain circulation leading to brain infarction, accounts for more than two-thirds of strokes [8,9,10]. Current state-of-the-art therapy includes intravenous thrombolysis (IVT) with alteplase or tenecteplase and mechanical thrombectomy (MT), alone or in combination with so-called bridging IVT [10,11,12,13]. Following large clinical trials, MT, particularly when combined with IVT, has become the new standard of care in patients with large vessel occlusion (LVO)-related acute stroke, as it results in better functional outcomes and a higher degree of angiographic revascularization, without a significant difference in the incidence of symptomatic intracerebral hemorrhage (sICH) or death, compared to IVT alone [14,15,16,17,18,19]. Nevertheless, it is imperative to acknowledge that certain patients may exhibit unfavorable prognoses or succumb shortly after the intervention. Therefore, it is of the highest interest to investigate whether there are a priori features of patients that should be assessed, treated, or modified before the procedure, which could be relevant to later adverse outcomes.

In previously published work, the estimated rate of in-hospital mortality in stroke ranges from 11 to 15% [20]. The percentage of deaths escalate further if 90-day mortality is included, reaching as high as 27%, with 52% of all death cases occurring during the first week after the MT treatment [21]. About 2/3 of in-hospital deaths are attributed to non-modifiable, baseline factors such as age and baseline stroke severity, while post-stroke complications determine the remaining third [20,22]. In practice, a degree of independence prior to the stroke measured with the premorbid modified Rankin scale (pre-mRS) is an important determining factor in whether MT is going to be performed. It is a 7-point scale where 0 signifies no symptoms, 4 indicates mobility only with assistance from another person, and 6 denotes death [23]. Stroke patients with a pre-mRS score of 4 or 5 before the onset of symptoms are generally not considered for mechanical thrombectomy and are excluded from most trials on endovascular treatment (EVT) [24]. However, for stroke patients with a pre-mRS score of 0–3, mechanical thrombectomy should not be withheld based solely on other features. Nevertheless, in an ideal scenario, having a reliable predictive tool before intervention could enable stakeholders to anticipate potential outcomes better.

Machine learning (ML) has become apparent as a competent predictive tool in many medical fields since, through modeling, it can handle complex relationships in data, uncover subtle information, and use these insights to automatically generate and summarize new knowledge [25]. This cannot be said for classical statistical approaches, as the increase in data complexity may make classical statistical inference less tractable [26,27]. In the currently available literature, ML models have shown superiority when compared to the standard statistical and clinical prediction models [28], and since there is increasing complexity, numerosity, and multifactoriality of available data sets [29], interest in their usage is rapidly increasing. On the other hand, clinicians can still feel confused and uncertain about the use of these models in everyday practice. The ML’s “black box” represents a major barrier to this progress since an understanding of a model’s decision-making process is crucial for its implementation in the clinical environment [30]. This concern could play an important role in daily interactions with patients, because, without an explanation of how a diagnosis, prognosis, or treatment plan was made, a lack of trust can be expected [31]. Fortunately, significant progress has been accomplished within the last years by developing interpretation methods, such as the Sharpley Additive Explanation (SHAP) and Local Interpretable Model-Agnostic Explanations (LIMEs), which show the potential in helping with the interpretational riddle on both feature and individual levels. To the best of our knowledge, interpretable machine learning (IML) models and their role in the prediction of in-hospital death after anterior circulation MT in AIS patients are still an unexplored research area.

In this research, we aimed to develop and internally validate IML models that would predict the probability of in-hospital death after anterior circulation MT in LVO-AIS patients. We hypothesized that ML would generate an easily understandable predictive model, highlighting the key features contributing to the unfavorable outcome and offering a better understanding of this complexity. The built model could be used to identify AIS patients less likely to benefit from recanalization and patients with a high risk of dying after the procedure.

## 2. Materials and Methods

### 2.1. Analyzed Group and Data Processing

This retrospective study analyzed the clinical data of consecutive patients with anterior circulation LVO-related ischemic stroke, treated with mechanical thrombectomy (MT) at the tertiary university middle-volume endovascular treatment center, Christian-Doppler-Klinik (Salzburg, Austria). The local ethics committee approved the retrospective collection of data. The data were collected from the clinical information system during the analyzed period of 9 years (2012–2020), and the inclusion criteria were that (I) the patient was older than 18 years; (II) MT was used as a therapeutic approach; (III) occluded vessel(s) were in the anterior circulation of the brain; and additionally to be included into the in-hospital death (IHD) group, (IV) if a patient died, it occurred due to the nature of stroke or stroke-related complications [22,32] during the time of hospitalization. On the contrary, patients with occlusion of the posterior circulation were excluded from the study, and patients who died after the period of in-hospital stay were not included in the IHD group.

The gathered data were divided into several subcategories, based on their similarities (Table 1), and were analyzed on three levels: (I) the whole group; (II) the in-hospital death group (miserable outcome); and (III) the in-hospital survival group (favorable outcome). Python 3.10.9, provided by Anaconda, Inc. (Austin, TX, USA), was the programming language used in this research. In the first steps of preprocessing, variables from the data were screened for missing values, and if this number went beyond 20%, they were excluded from any further analysis. A categorization of the variables followed, in which the division was made into categorical and continuous groups. Missing values were imputed using the most common value method for categorical variables and the median value for continuous ones.

Following a prior procedure, the data were divided into the outcome (target variable) and the input features. Outcome was represented by the parameter ‘in-hospital death’, which included two options: (I) *miserable outcome*, if a patient died during an in-hospital stay, and (II) *favorable outcome* if a patient survived. In this research, the terms in-hospital death and miserable outcome were alternately used to describe the same result, as we wanted to differentiate it from the unfavorable, or poor outcome, which could refer to different degrees of disability and therefore diminish the significance of the issue. The input features consisted of 53 variables, which were statistically compared between the two possible outcomes (Supplementary Table S1). Categorical features were expressed as numbers (percentages), and the chi-squared test was used to determine differences between the two outcome groups. Continuous features were presented as medians (interquartile range) and analyzed by the Student *t*-test or Mann–Whitney U test, based on sample normality. Two-tailed tests were used, and statistical significance was observed at level *p* < 0.05 for every variable. To address the collinearity between features, a correlation analysis was performed. We used the chi-squared test of independence for categorical features, presenting results as *p* values, with a statistically significant correlation being *p* < 0.05. The Pearson and Spearman Correlation coefficients were calculated for continuous features, based on their distribution, representing results as coefficients ranging from −1 to +1, presenting the strength and direction of the linear relationship between two continuous variables.

Data normalization followed, which is one of the first preprocessing tasks to be performed during analysis [33], and it implies that the data are transformed or scaled so that an equal contribution of each feature is achieved. This was carried out by Z-score normalization, in which the values of a feature are normalized based on the mean (average) value and standard deviation, as this process reduces numerical instabilities between the analyzed features [34,35].

### 2.2. Two-Step Feature Selection Process

After the previous steps, variables were divided into two categories: (I) *Baseline features* (*n* = 42) that were gathered or present during the patient’s admission, and (II) *Intervention-related features* (*n* = 11) collected or calculated after the endovascular treatment. As the input features included 53 variables, the feature selection technique was applied as it enables the selection of those features that contribute the most to the prediction variable or the outcome in machine learning (ML) algorithms. It is primarily carried out to remove unimportant features, improve analysis efficacy, and adapt the data set to best suit ML classifiers [36]. First, irrelevant features were removed after the univariate analysis, and then, redundant and useless features were excluded by a wrapper approach [37]. Univariate analysis was based on the features’ type. The Shapiro–Wilk normality test was conducted before the continuous variables analysis [38], and as asymmetric distribution was observed, the Mann–Whitney-Wilcoxon test was applied [39], while the chi-squared test was used in the categorical variables analysis [40]. After the removal of the irrelevant features, Recursive Feature Elimination (RFE) was used, as a method of wrapper models [41]. This algorithm eliminates one backward feature during one iteration and prevents information loss while showing high classification performances when compared to many other feature reduction methods [42]. This algorithm was used with four different classifiers—logistic regression (LG), random forest (RF), gradient boosting (GB), and extreme gradient boosting (XGB)—and the number of possible features to be included in the classification was not limited, and it solely depended on the model. The RFE process was performed for every classifier separately, and two features’ sets were gained: (I) *Pre-MT set*, including only baseline features, and (II) *Post-MT set*, which included both baseline and intervention-related variables.

To statistically address the influence of chosen variables on the miserable outcome, binomial regression analysis was performed, calculating the odds ratios and plotting the estimated marginal means plots. The most optimal cut-off values to predict the miserable outcomes were determined based on sensitivity and specificity.

### 2.3. Data Sampling and Construction of Two Predictive Models

As the outcome group was unbalanced (miserable outcome, *n* = 133 vs. favorable outcome, *n* = 469), we performed a data sampling technique to reduce the disparity. There are two basic sampling techniques, including random oversampling (ROS) and random undersampling (RUS). Despite the lack of conclusive evidence concerning the superiority of any approach, it has been reported that oversampling may lead to overfitting of the model [43,44,45,46], which is why we chose the RUS as more appropriate. In this approach, the majority classes are randomly eliminated, so that equal distribution can be achieved [47].

**Figure 1:**
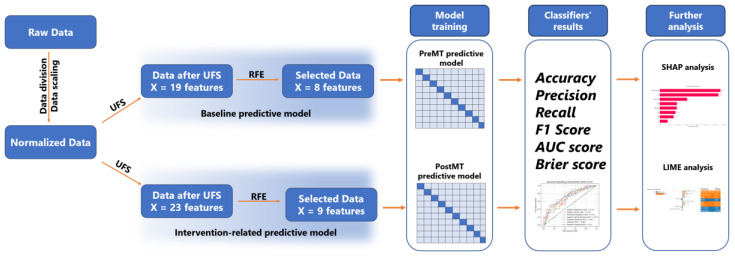
Summarized steps of the analysis. After a two-step feature selection process, with univariate feature selection (UFS) as the first step and Recursive Feature Elimination (RFE) as the second, models were trained, evaluated, and tested. After obtaining the classifiers’ results, features and individual level interpretations were performed for the best-performing classifiers in both models, using SHAP and LIME.

By random splitting, data were divided into three groups: (I) a *Training set* that included 70% of the input data, (II) an *Evaluation set* consisting of 15%, and (III) a *Test set* comprising 15% of the original data. Using Python’s Scikit-learn library, four classifiers were trained, internally evaluated, and tested: logistic regression (LR), random forest (RF), gradient boosting (GB), and extreme gradient boosting (XGB). As previously mentioned, two predictive models were created for every classifier based on the input features’ sets: (I) *Pre-MT model*, and (II) *Post-MT model*. During the training, each classifier used RFE-preselected feature sets, and the training included 10-fold cross-validation, during which the training set was divided into 1 validating and 9 training subsets (Figure 1). After the internal evaluation and testing, results were expressed through evaluation metrics such as accuracy, precision, recall, F1 score, and the area under the receiver operating characteristic (AUC-ROC) curve. Since the AUC has been proven to be a better measure than accuracy in the learning algorithm evaluation [48], it was used as a measure of discrimination [49], while the model calibration was expressed through calibration curves and Brier scores, where lower scores reflected better model calibration [50].

### 2.4. Interpretable Framework-Sharpley Additive Explanation (SHAP) and Local Interpretable Model-Agnostic Explanations (LIME)

Best-performing classifiers, based on the AUC value, were more thoroughly analyzed through Sharpley Additive Explanation (SHAP) values. This locally interpretable explanatory method can rationalize machine learning (ML) algorithm predictions, regardless of their complexity, as it identifies and maps features that reduce or increase the probability of the predicted outcome [51]. Additionally, SHAP can help with the understanding of features’ importance and, therefore, enhance their clinical interpretability [25]. Features were presented based on their order of importance (Absolute Mean SHAP Plot) in the predictive model.

To better understand the classification-making process on an individual level, Local Interpretable Model-Agnostic Explanations (LIMEs) were applied, as they can extract the contribution of key features during it [52]. This analysis was performed for both models, randomly choosing a patient from both groups. This figure (Figure 5)/Figure 5. shows the overall predicted probability of in-hospital death and favorable outcome on the left, classification details in the middle, and feature values and categories on the right. Continuous features were presented with their true values, while the categorical features were labeled based on the included category.

## 3. Results

### 3.1. Analyzed Group

A total of 602 patients fulfilled the inclusion criteria. In our cohort, around 54% (*n* = 328) of all patients were women, 133 (22%) had miserable outcomes, and the median age was 76 years (interquartile range (IQR) 65–83 years). Statistical feature comparison is presented in Table 1, and the correlation matrices are stored in Supplementary Table S2 for categorical features and Supplementary Table S3 for continuous ones. Out of all baseline variables, age (81 (IQR 74–87) vs. 74 (IQR 62–82), *p* < 0.001), baseline NIHSS value (20 (IQR 16–24) vs. 17 (IQR 11–21), *p* < 0.001), and peripheral artery disease (19/133 (14%) vs. 14/469 (3%), *p* < 0.001) showed the most statistically significant disparity between the two analyzed groups, while the most significant intervention-related feature was the ‘Number of steps’ (3 (IQR 2–4) vs. 2 (IQR 1–4), *p* = 0.011). Correlation analysis showed high collinearity between the previous stroke, the presence of peripheral arterial disease, and higher pre-mRS values.

### 3.2. The Results of a Two-Step Feature Selection Process

During a two-step process, variables for the predictive models were chosen as a product of univariate feature selection (UFS) and Recursive Feature Elimination (RFE). In the baseline set, those 8 features were age, baseline NIHSS value, neutrophil-to-lymphocyte ratio (NLR), international normalized ratio (INR), the type of the affected vessel (‘Vessel type’), peripheral arterial disease (PAD), baseline glycemia, and premorbid modified Rankin scale (pre-mRS). In the intervention-related set, the RFE method did not include pre-mRS, but it added two features: puncture-to-the-procedure end time (PET), and onset-to-puncture time (OPT). The RFE-selected sets were used in further analysis. The whole process is summarized in Figure 1.

**Figure 2:**
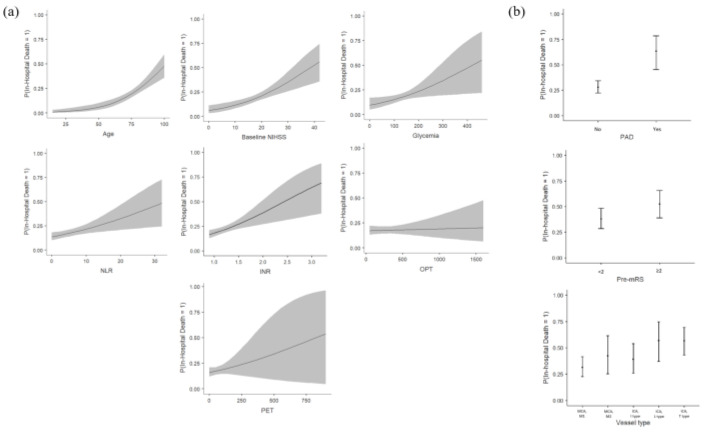
Estimated marginal means graphs of chosen features. Continuous (**a**) and categorical (**b**) features have been presented in the graph. Continuous features are represented with their true values, and categorical based on the analyzed categories. Estimated marginal means of continuous features are presented with a solid line, representing the predicted probability of in-hospital death based on the respective variable shown on the x-axis. The gray shaded areas depict the 95% confidence intervals around the estimated marginal means, with a wider gray area indicating more uncertainty and a narrower gray area indicating less uncertainty about the estimate. NIHSS, National Institutes of Health Stroke Scale; NLR, neutrophil-to-lymphocyte ratio; INR, international normalized ratio; OPT, onset-to-puncture time; PET, puncture-to-end time; PAD, peripheral arterial disease; pre-mRS, premorbid modified Rankin scale; MCA, middle cerebral artery; ICA, internal carotid artery.

Binomial logistic regression results are presented in Table 2, showing the influence of chosen features on in-hospital death (IHD) occurrence. Out of continuous features, INR had the highest impact with the most optimal cut-off value of 1.29, and an increase of 0.2 led to 37.2% higher chances of IHD. One unit increase in baseline NIHSS increased the risk of IHD by approximately 9%, with the optimal cut-off value of 20, one unit of NLR (the optimal cut-off = 3.8) as well as 10 mg/dL of blood glucose (the optimal cut-off = 120 mg/dL) by 7%, and one year of age (the optimal cut-off value = 78 years) by 6%. Selected time frames showed a lower influence on IHD, where every 10 min of OPT (the optimal cut-off value = 285 min) increased risk by 1% and 10 min of PET (the optimal cut-off value = 54 min) by 2%. The cut-off values tables are stored in Supplementary Table S4. Categorical variables showed high influence, as the presence of peripheral arterial disease (PAD) increased the chances of developing IHD 3.5 times and a premorbid mRS value of 2 or higher by 80%. The results are plotted in Figure 2.

### 3.3. Predictive Models Evaluation

**Figure 3:**
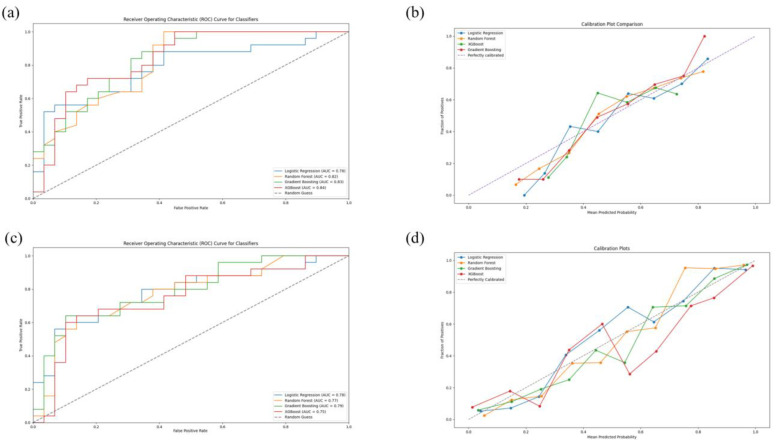
Evaluation of the predictive models. The receiver operating characteristic curves (ROCs) for pre-MT (**a**) and post-MT (**c**) models. Random guessing is represented by the black dashed line, with an AUC of 50% (0.5). The calibration plots with reliability curves for pre-MT (**b**) and post-MT (**d**) classifiers are also visualized, with the black dashed line representing perfect calibration.

After the training, internal evaluation, and testing, the performances of the classifiers were expressed through the previously mentioned parameters (Table 3). Their ROC curves and the calibration plot with reliability curves are visualized in Figure 3. For the Pre-MT model, gradient boosting (GB) showed itself as the best-performing classifier, during all phases, with an AUC of almost 80% (0.7903) and the lowest Brier score of 0.2009. The post-MT classifier evaluation showed that the extreme gradient boosting (XGB) had the highest performance with an AUC score of 84% (0.8372) and a Brier score of 0.1194.

#### 3.3.1. SHAP Analysis–Feature Level Interpretation

**Figure 4:**
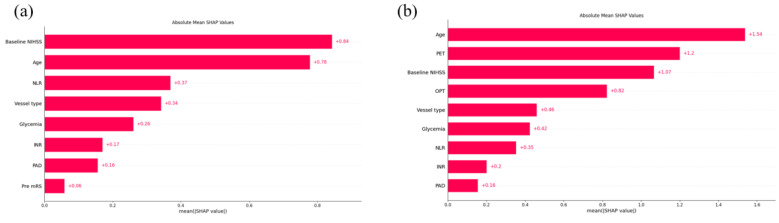
The SHAP analysis. The SHAP analysis graphs for in-hospital death prediction for the Pre-MT (**a**) and the Post-MT (**b**) models were visualized as absolute mean SHAP values. NIHSS, National Institutes of Health Stroke Scale; NLR, neutrophil-to-lymphocyte ratio; INR, international normalized ratio; PAD, peripheral arterial disease; pre-mRS, premorbid modified Rankin scale; OPT, onset-to-puncture time; PET, puncture-to-end time.

For the best-performing classifier, in both models, SHAP values were calculated, and the order of importance is presented in Figure 4. Based on the SHAP analysis of the Pre-MT model (Figure 4a), the most influential features that increase the risk of developing a miserable outcome were baseline NIHSS, age, NLR, glycemia, and INR. In addition, pre-mRS of two or higher, internal carotid artery occlusions (type L and T ICA terminus occlusions), and present peripheral arterial disease were also important contributing factors. The SHAP analysis of the XGB classifier (Figure 4b) shows that the same factors as previously mentioned influence the outcome prediction, with the addition of longer ‘Puncture-to-end time’ (PET) and ‘Onset-to-the-puncture time’ (OPT).

#### 3.3.2. LIME Analysis–Individual Level Interpretation

**Figure 5:**
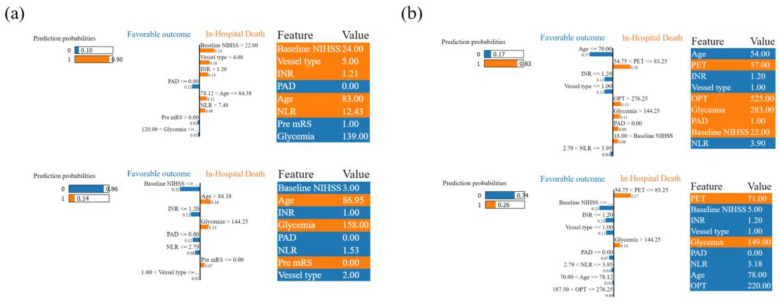
The LIME analysis. Local Interpretable Model-Agnostic Explanations (LIME) plot from the testing set of the GB (**a**) and XGB (**b**) models. True positive (upper examples) and true negative (lower examples) patients were chosen for the interpretation. NIHSS, National Institutes of Health Stroke Scale; INR, international normalized ratio; PAD, peripheral arterial disease; NLR, neutrophil-to-lymphocyte ratio; pre-mRS, premorbid modified Rankin scale; OPT, onset-to-puncture time; PET, puncture-to-end time.

The Pre-MT model’s LIME analysis is visualized in Figure 5a, simplifying the GB classifier’s decision-making algorithm, and Figure 5b visualizes the post-MT model, based on the prediction of XGB. Continuous features are presented with their true values, while the categorical features are labeled as follows. pre-mRS category 0 corresponds to a score of 2 or higher, peripheral arterial disease (PAD) category 1 represents the presence of the disease, while the vessel type is associated with the division in Table 1, within the 5 categories (1, M1 segment of the middle cerebral artery; 2, M2 segment of the middle cerebral artery; 3, internal carotid artery occlusion–type I; 4, internal carotid artery occlusion–type L; 5, internal carotid artery occlusion–type T). In chosen patients, probability scores, based on eight features in the Pre-MT model, which is nine in the Post-MT one, predict both favorable and miserable outcomes.

## 4. Discussion

To the best of our knowledge, this is the first study that utilizes interpretable machine learning models for the in-hospital mortality analysis of patients who underwent thrombectomy for anterior circulation occlusive stroke. Four classifiers were trained, internally evaluated, and tested for both models. The two best-performing classifiers were the gradient boosting (GB) for the Pre-MT model and the extreme gradient boosting (XGB) for the Post-MT predictive model, which included both baseline and intervention-related parameters. Based on eight variables, GB achieved an AUC score of almost 80%, with a Brier score of 0.2, while the XGB accomplished better performances, an AUC score of 84%, and a Brier score of 0.12, using nine features. In addition to seven identical features that were incorporated into both models (age, baseline NIHSS value, neutrophil-to-lymphocyte ratio (NLR), international normalized ratio (INR), the type of the affected vessel, baseline glycemia, and the presence of peripheral arterial disease), the baseline model also included premorbid modified Rankin scale (pre-mRS), while the second model involved puncture-to-the-procedure-end time (PET) and onset-to-puncture time (OPT). We aimed to more thoroughly explain the “black box” of ML models through Sharpley Additive Explanation (SHAP), which interpreted the model at the feature level, and Local Interpretable Model-Agnostic Explanations (LIMEs), used for the interpretation at the individual level.

### 4.1. Baseline Features Prior to Thrombectomy

In our baseline, Pre-MT predictive model, baseline NIHSS value and age stood out as the most significant features, which is similar to the previous research [20,22]. Out of all analyzed laboratory findings, the neutrophil-to-lymphocyte ratio followed by baseline glycemia and INR value have proven themselves as the most appropriate for mortality prediction.

Hyperglycemia was linked to unfavorable clinical outcomes and mortality, in patients with large vessel occlusion (LVO) that were treated with the MT [53], which probably occurs as a consequence of metabolic alterations, such as intracellular acidosis, decreased mitochondrial function, and increased levels of reactive oxygen species (ROS), leading to neuronal damage [54]. In a previously published study, in which interpretable machine learning was used to predict the development of 24 h early neurological deterioration (END) in MT-treated ischemic stroke patients, hyperglycemia was the most important feature that contributed to the unfavorable outcome [25]. Even though hypoglycemia was proven to predict higher 90-day mortality in MT-treated patients [54], it did not demonstrate itself as significant in this analysis. A second laboratory feature, the neutrophil-to-lymphocyte ratio (NLR), was also linked with the stroke outcome. Higher NLR was associated with a more common death, unfavorable, or poor 90-day outcome in ischemic stroke patients who underwent reperfusion therapy [55,56]. Accompanied by hyperglycemia, which is an important factor for the development of thromboinflammation [57], elevated NLR represents a response to the inflammatory nature of stroke, which is a major contributor to its pathobiology and outcome [58]. Although MT can be safely and effectively performed in emergent large vessel occlusion (ELVO) patients with high INR [59], these patients are more prone to worse clinical outcomes and higher mortality risk [60], which was also found in our study.

Taking into consideration occluded vessels, our analysis has shown that the T-type internal carotid occlusion was mostly associated with in-hospital death, while the isolated middle cerebral artery (MCA) occlusion, specifically in the M1 segment, was more abundant in the favorable outcome group. In the available literature, no research has explicitly analyzed the connection between the occlusion type and in-hospital mortality. However, it was found that the internal carotid artery (ICA) occlusion is a significant predictor of 90-day mortality [61] and that the L- and T-types of terminal carotid occlusion were more frequent in the 90-day mortality group [62]. Outcomes of the thrombectomy of the MCA M1 and M2 segments showed that the 90-day functional outcomes did not differ between segments [63]. Although peripheral artery disease showed no association with the 90-day functional or safety outcomes in a large cohort study [64], it certainly is a strong independent predictor for stroke [65], and in our analysis, it was an important contributing factor to the miserable outcome.

It is estimated that only around 20% of the pre-stroke-disabled patients (mRS > 2) return to their premorbid values after the MT, and, additionally, a good outcome is less frequent, while the mortality and risk of symptomatic intracranial hemorrhage (sICH) are higher [66,67,68,69]. Based on the previous information, higher premorbid mRS could be classified as an important unfavorable factor, which was also chosen by our feature selection process.

### 4.2. Features Pertinent to Mechanical Thrombectomy

Similar to the previous model, in previously published works, most of the intervention-related features have shown significance in the 90-day functional outcome and survival prediction, but their role in the context of in-hospital mortality has not been analyzed. It is shown that shorter onset-to-puncture (OPT) is associated with a lower degree of 3-month disability and higher independence at discharge [70,71,72,73], and besides long-term outcomes, in this research, it was shown that the longer OPT is an important predictor of in-hospital death. Other than this, the feature selection model also included PET, which showed even higher predictive capabilities, compared to the OPT. In the available literature, this parameter is associated with 3-month mortality, especially if it exceeded 60 min, and post-interventional complications if this period went beyond 35 min. Possible explanations for this occurrence might be that the longer procedure duration usually happens when there is an occlusive material resistant to extraction. Besides this, an extended intervention timeframe can burden compensatory collaterals and lead to irreversible injuries [72,73,74]. Even though these factors individually demonstrated low predictive power, they collectively contributed to the model’s overall predictions. This highlights the importance of analyzing multiple features simultaneously rather than isolated, a task that is effectively performed by machine learning models.

There are several study limitations. First, all included patients died due to stroke, or its complications, but the precise cause of death was not more thoroughly differentiated in the research. As some of the most common conditions that contribute to in-hospital mortality are respiratory infections and brain edema [20,22], future studies may focus on these as an outcome and not the overall in-hospital mortality of ischemic stroke. Second, in our study, we used a hybrid feature selection approach, which is just one of many techniques that can be used during this preprocessing phase. It means that a variety of different methods could potentially be applied to this phase in the forthcoming studies, so that their influence on the results may become a subject of the analysis. Third, regardless of the high predictive power, for the evaluation and implementation of tested models, a higher number of included patients is necessary. Finally, this study only included patients with anterior circulation ischemic stroke, so it does not apply to the posterior vasculature.

## 5. Conclusions

In this study, two supervised ML models were constructed to predict in-hospital death after the anterior circulation MT, in large-vessel-occlusion-related acute ischemic stroke patients. The constructed models achieved good predictive performances, and the prediction-making process was more thoroughly understood with the usage of SHAP and LIME. The baseline parameters showed the strongest impact on the miserable outcome prediction, but the highest predictive power was obtained when time frames, OPT and PET, were also included in the model. We found that baseline predictors NIHSS and age, neutrophil-to-lymphocyte ratio, followed by baseline glycemia and INR value prove to be the most appropriate for mortality prediction. In addition, a longer time elapsed from arterial puncture to the end of the intervention was also associated with higher mortality. The majority of features related to endovascular treatment did not show an essential role in the miserable outcome prediction, since factors like the success of recanalization (TICI) and intervention-related complications demonstrated low or almost no impact on in-hospital death prediction.

## Figures and Tables

**Table 1:** Whole sample analysis.

Variables	All Patients (N = 602)	Patients with the Miserable Outcome (N = 133)	Patients with the Favorable Outcome (N = 469)	*p* Value
Sociodemographic data				
Age, median (IQR)	76 (65–83)	81 (74–87)	74 (62–82)	<0.001
Sex, *n* (%)				0.113
Female	328/602 (54%)	81/133 (61%)	247/469 (53%)	
Male	274/602 (46%)	52/133 (39%)	222/469 (47%)	
Days to In-hospital death (IQR)		6 (2.5–14)		
Vascular risk factors and comorbidities				
Previous stroke, *n* (%)	92/602 (15%)	32/133 (24%)	60/469 (13%)	0.002
Peripheral arterial disease (PAD), *n* (%)	33/602 (5%)	19/133 (14%)	14/469 (3%)	<0.001
Tobacco smoking *n* (%)	381 (63%)	81 (61%)	300 (64%)	0.393
Atrial fibrillation, *n* (%)	237/602 (39%)	62/133 (47%)	175/469 (37%)	0.066
Diabetes mellitus, *n* (%)	90/602 (15%)	26/133 (20%)	64/469 (14%)	0.122
Arterial hypertension, *n* (%)	433/602 (72%)	103/133 (77%)	330/469 (70%)	0.135
Internal carotid artery (ICA) stenosis, *n* (%)	86/602 (14%)	24/133 (18%)	62/469 (13%)	0.206
Ischemic heart disease, *n* (%)	110/602 (18%)	31/133 (23%)	79/469 (17%)	0.115
Kidney failure, *n* (%)	53/602 (9%)	22/133 (17%)	31/469 (6%)	0.001
Heart failure, *n* (%)	75/602 (12%)	22/133 (17%)	53/469 (11%)	0.142
Statins usage, *n* (%)	149/602 (25%)	31/133 (23%)	118/469 (25%)	0.747
Antiplatelets drug usage, *n* (%)	158/602 (26%)	38/133 (29%)	120/469 (26%)	0.563
Anticoagulants usage, *n* (%)	58/602 (10%)	24/133 (18%)	34/469 (7%)	<0.001
Direct oral anticoagulants usage, *n* (%)	57/602 (9%)	20/133 (15%)	37/469 (8%)	0.02
Baseline laboratory values				
C-reactive protein (CRP) (mg/dL), median (IQR)	0.39 (0.18–0.91)	0.42 (0.19–1.48)	0.39 (0.17–0.81)	0.008
Glycemia (mg/dL), median (IQR)	120 (107–143)	124 (112–157)	119 (106–141)	0.004
HbA1c (%), median (IQR)	5.7 (5.5–5.9)	5.7 (5.5–5.9)	5.7 (5.4–5.9)	0.167
Creatinine (mg/dL), median (IQR)	0.89 (0.77–1.08)	0.93 (0.82–1.25)	0.88 (0.76–1.05)	0.001
Cholesterol (mg/dL), median (IQR)	153 (134–177)	153 (132–158)	153 (134–181)	0.025
Triglycerides (mg/dL), median (IQR)	100 (79–129)	100 (81–122)	100 (78–133)	0.405
High-density lipoprotein (HDL) (mg/dL), median (IQR)	46 (39–53)	46 (40–49)	46 (39–54)	0.708
Low-density lipoprotein (LDL) (mg/dL), median (IQR)	95 (78–112)	95 (73–98)	95 (78–116)	0.002
White Blood Cells (WBC) (×10^9^/L), median (IQR)	8.59 (7.00–10.8)	8.59 (7.08–11.89)	8.59 (6.99–10.76)	0.107
Neutrophils (×10^9^/L), median (IQR)	6.27 (4.58–8.23)	6.27 (4.98–8.88)	6.02 (4.52–8.11)	0.005
Lymphocytes (×10^9^/L), median (IQR)	1.55 (1.10–2.10)	1.40 (0.91–1.97)	1.63 (1.15–2.11)	0.001
Red Blood Cells (RBC) (×10^12^/L), median (IQR)	4.41 (4.07–4.74)	4.41 (4.01–4.81)	4.41 (4.09–4.72)	0.447
Hematocrit (HCT) (%), median (IQR)	39.2 (36.0–42.2)	39.20 (35.3–42.4)	39.20 (36.20–42.20)	0.566
Platelets (PLT) (×10^9^/L), median (IQR)	224 (188–273)	224 (187–284)	224 (188–269)	0.368
Fibrinogen (mg/dL), median (IQR)	341 (286–399)	359 (315–446)	341 (277–394)	0.002
International normalized ratio (INR), median (IQR)	1.20 (1.20–1.20)	1.20 (1.20–1.21)	1.20 (1.20–1.20)	0.009
Neutrophil-to-lymphocyte ratio (NLR), median (IQR)	4.06 (2.41–6.50)	3.95 (2.86–8.67)	3.95 (2.29–5.86)	0.001
Systemic inflammatory index (SII), median (IQR)	908 (511–1522)	919 (618–1951)	918 (494–1395)	0.006
Clinical data				
pre-mRS ≥ 2, *n* (%)	91/602 (15%)	32/133 (24%)	59/469 (13%)	0.002
Baseline NIHSS, median (IQR)	18 (12–22)	20 (16–24)	17 (11–21)	<0.001
Wake-up stroke, *n* (%)	114/602 (19%)	32/133 (24%)	82/469 (17%)	0.113
ASPECTS > 6, *n* (%)	536/602 (89%)	116/133 (87%)	420/469 (90%)	0.546
TOAST				
Cardioembolic cause (CE), *n* (%)	331/602 (55%)	86/133 (65%)	245/469 (52%)	0.015
Large artery atherosclerosis (LAA), *n* (%)	81/602 (13%)	12/133 (9%)	69/469 (15%)	0.120
Other, or unknown cause, *n* (%)	190/602 (32%)	35/133 (26%)	155/469 (33%)	0.171
Thrombolysis, *n* (%)	361/602 (60%)	64/133 (48%)	297/469 (63%)	0.002
Occluded vessel and leptomeningeal collaterals				
Vessel type, *n* (%)				
Middle Cerebral Artery-M1 segment	335/602 (56%)	55/133 (41%)	280/469 (60%)	<0.001
Middle Cerebral Artery-M2 segment	47/602 (8%)	11/133 (8%)	36/469 (8%)	0.966
Internal Carotid Artery-I type	93/602 (15%)	20/133 (15%)	73/469 (16%)	0.990
Internal Carotid Artery-L type	33/602 (5%)	12/133 (9%)	21/469 (4%)	0.069
Internal Carotid Artery-T type	94/602 (16%)	35/133 (26%)	59/469 (13%)	<0.001
Vessel side, *n* (%)				0.567
Right	287/602 (48%)	60/133 (45%)	227/469 (48%)	
Left	315/602 (52%)	73/133 (55%)	242/469 (52%)	
Leptomeningeal collaterals (LC), *n* (%)				0.001
Good or Equal to the unaffected side	454/602 (75%)	85/133 (64%)	369/469 (79%)	
Absent	148/602 (25%)	48/133 (36%)	100/469 (21%)	
Endovascular procedure information				
Onset-to-puncture time (OPT)	192 (154–270)	200 (152–329)	195.0 (140–285)	0.181
Puncture-to-end time (PET), median (IQR)	52 (38–66)	62 (54–90)	55 (33–79)	0.358
Onset to the procedure end time (OPET)	264 (219–318)	276 (237–389)	269 (205–343)	0.269
Number of steps, median (IQR)	2 (1–3)	3 (2–4)	2 (1–4)	0.011
Procedure type, *n* (%)				
Failed/Abandoned thrombectomy, *n* (%)	55/602 (9%)	11/133 (8%)	44/469 (9%)	0.824
Aspiration only	229/602 (38%)	41/133 (31%)	188/469 (40%)	0.066
Stent-retriever only	24/602 (4%)	5/133 (4%)	19/469 (4%)	1.000
Aspiration and stent-retriever	294/602 (49%)	76/133 (57%)	218/469 (46%)	0.038
Procedure complications, *n* (%)				
No complications	586/602 (97%)	129/133 (97%)	457/469 (97%)	1.000
Downstream complications	11/602 (2%)	3/133 (2%)	8/469 (2%)	0.959
Distal complications	5/602 (1%)	1/133 (1%)	4/469 (1%)	1.000
Vessel perforation	45/602 (7%)	8/133 (6%)	37/469 (8%)	0.590
Bleeding type, *n* (%)				
No bleeding	415/602 (69%)	85/133 (64%)	330/469 (70%)	0.189
Symptomatic bleeding	18/602 (3%)	1/133 (1%)	17/469 (4%)	0.153
Any other bleeding	169/602 (28%)	47/133 (35%)	122/469 (26%)	0.045
Thrombolysis in Cerebral Infarction (TICI), *n* (%)				0.040
Incomplete recanalization (TICI = 0-2a)	152/602 (25%)	24/133 (18%)	128/469 (27%)	
Complete recanalization (TICI = 2b-3)	450/602 (75%)	109/133 (82%)	341/469 (73%)	
Internal carotid artery stenting, *n* (%)	34/602 (6%)	11/133 (8%)	23/469 (5%)	0.203
Osteoclastic decompressive craniectomy, *n* (%)	29/602 (5%)	8/133 (6%)	21/469 (4%)	0.616

IQR, interquartile range; mRS, modified Rankin scale; NIHSS, the National Institutes of Health Stroke Scale; ASPECTS, Alberta stroke program early CT score; TOAST, Trial of ORG 10,172 in Acute Stroke Treatment.

**Table 2:** Binomial logistic regression results of the chosen parameters associated with in-hospital death after mechanical thrombectomy for anterior large vessel occlusion stroke, N = 602.

Predictor	Estimate	95% Confidence Interval		SE	z	*p* Value	Odds Ratio	95% Confidence Interval	
		Lower	Upper					Lower	Upper
Age	0.053	0.034	0.074	0.010	5.289	<0.001	1.061	1.034	1.076
Baseline NIHSS	0.071	0.037	0.105	0.017	4.104	<0.001	1.086	1.038	1.111
Glycemia	0.005	0.000	0.009	0.002	2.317	0.020	1.007	1.001	1.009
NLR	0.055	0.016	0.095	0.020	2.736	0.006	1.071	1.016	1.100
INR	1.050	0.398	1.710	0.334	3.150	<0.001	2.866	1.488	5.517
PAD	1.505	0.751	2.259	0.384	3.910	<0.001	4.504	2.120	9.569
pre-mRS	0.590	0.074	1.105	0.263	2.240	0.025	1.803	1.077	3.019
OPT	0.000	0.000	0.001	0.000	0.258	0.796	1.001	0.999	1.001
PET	0.002	0.001	0.006	0.002	1.049	0.294	1.002	0.998	1.006

SE, standard error; NIHSS, National Institutes of Health Stroke Scale; NLR, Neutrophil-to-lymphocyte ratio; INR, international normalized ratio; PAD, peripheral arterial disease; pre-mRS, premorbid modified Rankin scale; OPT, onset-to-puncture time; PET, puncture-to-end time.

**Table 3:** Evaluation of the classifiers associated with in-hospital death after mechanical thrombectomy for anterior large vessel occlusion stroke, N = 602.

Classifier		Accuracy	Precision	Recall	F1-Score	AUC-ROC	Brier Score
Pre-MT Model	LR	0.7222	0.7083	0.6800	0.6939	0.7821	0.2205
	RF	0.7222	0.7273	0.6400	0.6809	0.7648	0.2058
	GB	0.6667	0.6207	0.7200	0.6667	0.7917	0.2009
	XGB	0.7037	0.6800	0.6800	0.6800	0.7503	0.2182
Post-MT Model	LR	0.7222	0.7273	0.6400	0.6809	0.7848	0.1328
	RF	0.7073	0.6800	0.6800	0.6800	0.8145	0.1192
	GB	0.6852	0.6667	0.6400	0.6531	0.7931	0.1197
	XGB	0.7407	0.7200	0.7200	0.7200	0.8372	0.1194

MT, mechanical thrombectomy; LR, logistic regression; RF, random forest; GB, gradient boosting; XGB, extreme gradient boosting; AUC-ROC, the area under the receiver operating characteristic curve.

## Data Availability

All data generated or analyzed during this study are included in this published article.

## Supplementary Materials

The following supporting information can be downloaded at: https://www.mdpi.com/article/10.3390/diagnostics14141531/s1, Table S1: Breakdown of features used in the research; Table S2: Categorical features correlation; Table S3: Continuous features correlation; Table S4: Cut-off values for continuous features.
