# Supplementary material for: Predictors of In-Hospital Mortality after Thrombectomy in Anterior Circulation Large Vessel Occlusion: A Retrospective, Machine Learning Study
Source: Diagnostics (Basel). 2024 Jul 16;14(14):1531. doi: 10.3390/diagnostics14141531 (PMC11275350; doi:10.3390/diagnostics14141531)
Supplement: Supplementary file 1 [file diagnostics-14-01531-s001.zip › diagnostics-3088983-SI.pdf]

Supplementary Table S1. Breakdown of features used in the research.

In-hospital death (IHD)	Patients were divided into two groups:<br>a. Favorable outcome – patients who survived the in-hospital period, after anterior circulation mechanical thrombectomy<br>b. Miserable outcome – patients who died during the in-hospital period, due to stroke or post-stroke complications (20,22).	Categorical
Age		Continuous
Sex	Patients were divided into two categories:<br>a. Male<br>b. Female	Categorical
Days to In-hospital death	A period (in days) that passed from the stroke onset to the in-hospital death. It was calculated only for the patients in the Miserable outcome group.	Continuous
Previous stroke	Patients were divided into two categories:<br>a. Patients with a history of previous stroke<br>b. Patients without previous stroke	Categorical
Peripheral arterial disease	Patients were divided into two categories:<br>a. Patients with previous stroke<br>b. Patients without previous stroke	Categorical

Atrial fibrillation	Patients were divided into two categories:<br>a. Patients with registered atrial fibrillation during in-hospital stay, or available data about previously diagnosed AF<br>b. Patients without AF	Categorical
Diabetes mellitus	Patients were divided into two categories:<br>a. Patients with diagnosed DM<br>b. Patients without diagnosed DM	Categorical
Arterial hypertension	Patients were divided into two categories:<br>a. Patients with diagnosed AH<br>b. Patients without diagnosed AH	Categorical
Internal carotid artery (ICA) stenosis	Patients were divided into two categories:<br>a. Patients with observed significant ICA stenosis<br>b. Patients without significant ICA stenosis	Categorical
Ischemic heart disease	Patients were divided into two categories:<br>a. Patients with a history of IHD<br>b. Patients without a history of IHD	Categorical

Kidney failure (KF)	Patients were divided into two categories:<br><br>a. Patients with previously or newly diagnosed KF<br><br>b. Patients without KF	Categorical
Heart Failure	Patients were divided into two categories:<br><br>a. Patients with previously or newly diagnosed HF<br><br>b. Patients without HF	Categorical
Statins usage	Patients were divided into two categories:<br><br>a. Patients with previous usage<br><br>b. Patients without previous usage	Categorical
Antiplatelet drugs	Patients were divided into two categories:<br><br>a. Patients with previous usage<br><br>b. Patients without previous usage	Categorical
Anticoagulants usage	Patients were divided into two categories:<br><br>a. Patients with previous usage<br><br>b. Patients without previous usage	Categorical
Direct oral anticoagulants usage	Patients were divided into two categories:<br><br>a. Patients with previous usage<br><br>b. Patients without previous usage	Categorical

C-reactive protein (CRP)	mg/dL	Continuous
Glycemia	mg/dL	Continuous
HbA1c	%	Continuous
Creatinine	mg/dL	Continuous
Cholesterol	mg/dL	Continuous
Triglycerides	mg/dL	Continuous
High-density lipoprotein (HDL)	mg/dL	Continuous
Low-density lipoprotein (LDL)	mg/dL	Continuous
White Blood Cells (WBCs)	$\times 10^9$/L	Continuous
Neutrophils (Neu)	$\times 10^9$/L	Continuous
Lymphocytes (Ly)	$\times 10^9$/L	Continuous
Platelets (PLT)	$\times 10^9$/L	Continuous
Red Blood cells (RBC)	$\times 10^{12}$/L	Continuous
Hematocrit (HCT)	%	Continuous
Fibrinogen	mg/dL	Continuous
INR		Continuous
Neutrophil Lymphocyte ratio (NLR)	Calculated as the ratio between the neutrophil and lymphocyte number. $NLR = \frac{Neu}{Ly}$	Continuous
Systemic Inflammatory Index (SII)	Calculated as the division of the product of neutrophil and platelet number, and the number of lymphocytes. $SII = \frac{Neu * PLT}{Ly}$	Continuous
Pre mRS	Patients were divided into two groups: a. Patients with premorbid mRS value of 0-1 b. Patients with premorbid mRS value of 2 or more	Categorical

Baseline NIHSS		Continuous
Wake-up stroke	Patients were divided into two groups:<br>a. Patients with wake-up stroke, in which extensive neuroimaging, before MT, was performed<br>b. Patients without wake-up stroke	Categorical
ASPECTS	Patients were divided into two groups:<br>a. Patients with ASPECTS of 7 or more<br>b. Patients with ASPECTS of 6 or less	Categorical
TOAST	Patients were divided into three groups:<br>a. Patients with stroke caused by cardioembolism<br>b. Patients with stroke caused by large artery atherosclerosis<br>c. Patients with stroke of other or unknown cause	Categorical
Thrombolysis	Patients were divided into two groups:<br>a. Patients who have received IVT before MT treatment<br>b. Patients who didn’t receive IVT	Categorical

Type of the affected vessel	Based on the occluded vessel, patients were divided into 5 groups:<br>a. M1 segment of the middle cerebral artery<br>b. M2 segment of the middle cerebral artery<br>c. Type I occlusion of the internal carotid artery, or occlusion of the ICA only<br>d. Type L occlusion of the internal carotid artery, or occlusion of the ICA and MCA<br>e. Type T occlusion of the internal carotid artery, or occlusion of the ICA, MCA, and ACA	Categorical
Side	Patients were divided into three groups:<br>a. Left side<br>b. Right side	Categorical
Leptomeningeal collaterals	To evaluate leptomeningeal collaterals, we employed a grading system introduced by Angermaier et al (72). The authors categorize the vascular contrast filling into four categories: 0 = no collateral filling, 1 = collateral filling ≤ 50%, 2 = collateral filling > 50% but < 100%, and 3 = 100% collateral filling of the mismatch region. We collapsed categories into	Categorical

	good (categories 2 and 3) and poor (0 and 1). Based on the quantitative method, patients were divided into two groups:<br><br>a. Patients with equally visualized LC on both affected and unaffected sides of the brain and patients with unequal, but visualized LC on both affected and unaffected sides of the brain<br><br>b. Patients without or poorly visualized LC in the affected side of the brain	
Onset to puncture time	Time in minutes passed from the witnessed or self-reported onset of neurological symptoms to the moment when the catheter punctured the blood vessel to perform a mechanical thrombectomy.	Continuous
Onset to the procedure end time	Time in minutes passed from the witnessed or self-reported onset of neurological symptoms to the moment when a mechanical thrombectomy was completed.	Continuous
Puncture to end time	Time in minutes passed from the moment when the catheter punctured the blood vessel to perform a mechanical thrombectomy to the moment when a mechanical thrombectomy was completed.	Continuous
Number of steps	A number of passages through the culprit vessel needed to recanalize it.	Continuous

Procedure type	Patients were divided into 4 groups:<br><br>a. MT included only aspiration of the thrombotic mass<br><br>b. MT included the placement of the stent retriever<br><br>c. MT included both aspiration and placing of stent retriever<br><br>d. Failed/Abandoned thrombectomy - the occlusion site was inaccessible due to anatomical challenges or resistant occlusions, or passage of the thrombus was unsuccessful.	Categorical
Procedure complications	Patients were divided into three groups:<br><br>a. Patients without observed complications<br><br>b. Patients with distal complications, including pseudoaneurysm of femoral artery, big hematoma, and thrombotic occlusions of femoral or popliteal arteries.<br><br>c. Patients with downstream complications in the brain vasculature, including artery dissection, retention of part of the catheter in the blood vessel, or vessel perforation	Categorical
Vessel perforation	Patients were divided into two groups:<br><br>a. Patients with vessel perforation, as a distal	Categorical

	complication of mechanical thrombectomy<br><br>b. Patients without vessel perforation	
Osteoclastic decompressive craniectomy	Patients were divided into two groups:<br><br>a. Patients with performed osteoplastic decompressive craniotomy, due to elevated intracranial pressure<br><br>b. Patients without osteoplastic decompressive craniotomy	Categorical
Bleeding class	Patients were divided into three groups:<br><br>a. Patients without radiologically observed intracerebral bleeding<br><br>b. Patients with symptomatic intracerebral hemorrhage (sICH) – type 2 of the Heidelberg classification<br><br>c. Patients with observed bleeding types other than type 2 of the Heidelberg classification, including classes 1 and 3	Categorical

Thrombolysis in Cerebral Infarction (TICI) scale	Patients were divided into two categories:<br>1. Incomplete recanalization (TICI = 0-2a)<br>2. Complete recanalization (TICI =2b-3)<br><br>Five categories of the perfusion status after the performed intervention (10):<br><br>a. Grade 0: no perfusion<br><br>b. Grade 1: antegrade reperfusion past the initial occlusion, but limited distal branch filling with little or slow distal reperfusion<br><br>c. Grade 2a: antegrade reperfusion of less than half of the occluded target artery previously ischemic territory (e.g. in one major division of the (MCA) and its territory)<br><br>d. Grade 2b: antegrade reperfusion of more than half of the previously occluded target artery ischemic territory (e.g. in two major divisions of the MCA and their territories)<br><br>e. Grade 3: complete antegrade reperfusion of the previously occluded target artery ischemic territory, with the absence of visualized occlusion in all distal branches	Categorical

Internal carotid artery (ICA) stenting	Patients were divided into two groups:<br><br>a. Patients without ICA stenting<br><br>b. Patients with ICA stenting	Categorical

Supplementary table S2. Categorical features correlation

	Gender	Previous stroke	Peripheral artery disease	Atrial fibrillation	Diabetes mellitus	Arterial hypertension	Internal carotid artery stenosis	Ischemic heart disease	Kidney failure	Heart failure	Statins usage	Antiplatelet drugs	Anticoagulants usage	Direct oral anticoagulants usage	Wake-up stroke	Pre mRS	ASPECTS	TOAST	Thrombolysis	Vessel type	Vessel side	Leptomeningeal collaterals	Procedure type	Procedure complications	Vessel perforation	TICI	ICA stenting	Bleeding class
**Gender**		0,17	0,46	**0,02**	**0,01**	0,38	1,00	0,10	1,00	0,74	**0,00**	**0,02**	0,50	0,17	**0,04**	1,00	0,36	**0,00**	0,13	0,19	0,21	0,58	0,68	0,43	0,29	1,00	0,82	0,13
**Previous stroke**	0,17		**0,02**	0,05	**0,00**	**0,03**	1,00	0,32	0,12	1,00	**0,00**	**0,00**	**0,00**	**0,01**	0,57	**0,00**	0,08	0,07	**0,00**	0,53	0,52	0,25	0,71	0,29	0,51	0,74	0,43	0,09
**Peripheral artery disease**	0,46	**0,02**		0,19	0,14	0,10	0,16	**0,01**	0,39	0,12	0,30	0,16	0,79	0,11	0,51	**0,00**	0,35	0,66	0,07	0,49	0,96	0,30	0,95	0,62	0,69	0,86	**0,02**	0,64
**Atrial fibrillation**	**0,02**	0,05	0,19		0,25	**0,00**	0,87	**0,00**	**0,00**	**0,00**	0,19	**0,05**	**0,00**	**0,00**	0,68	**0,01**	0,83	**0,00**	**0,00**	0,07	0,45	**0,02**	0,24	0,34	0,69	0,77	0,64	0,56
**Diabetes mellitus**	**0,01**	**0,00**	0,14	0,25		**0,00**	**0,01**	**0,03**	0,14	0,09	**0,00**	**0,00**	1,00	1,00	0,44	**0,00**	1,00	0,62	**0,03**	**0,02**	0,40	0,38	**0,04**	0,27	0,33	0,50	1,00	0,23
**Arterial hypertension**	0,38	**0,03**	0,10	**0,00**	**0,00**		**0,00**	**0,00**	0,15	0,07	**0,00**	**0,00**	0,42	0,09	0,20	0,12	0,64	**0,00**	0,71	0,47	0,19	0,36	**0,01**	0,39	1,00	1,00	0,99	0,87
**Internal carotid artery stenosis**	1,00	1,00	0,16	0,87	**0,01**	**0,00**		0,33	0,17	0,68	**0,04**	**0,01**	0,25	0,75	0,53	0,48	0,44	**0,00**	1,00	0,15	**0,00**	0,93	0,50	0,30	**0,05**	0,54	0,70	0,51
**Ischemic heart disease**	0,10	0,32	**0,01**	**0,00**	**0,03**	**0,00**	0,33		**0,01**	**0,00**	**0,00**	**0,00**	0,73	**0,01**	0,39	**0,03**	0,71	**0,01**	0,10	0,86	0,72	0,39	0,88	0,45	0,61	0,19	0,68	0,52
**Kidney failure**	1,00	0,12	0,39	**0,00**	0,14	0,15	0,17	**0,01**		**0,00**	**0,04**	0,06	0,23	1,00	0,69	0,13	0,85	**0,04**	0,39	0,19	0,14	0,71	0,17	0,40	0,29	0,81	0,25	0,41
**Heart failure**	0,74	1,00	0,12	**0,00**	0,09	0,07	0,68	**0,00**	**0,00**		0,16	**0,02**	0,61	0,57	0,52	0,12	0,61	**0,00**	0,75	0,17	0,07	0,44	0,08	0,41	0,14	0,34	1,00	0,51
**Statins usage**	**0,00**	**0,00**	0,30	0,19	**0,00**	**0,00**	**0,04**	**0,00**	**0,04**	0,16		**0,00**	0,42	0,63	0,10	0,59	0,35	0,89	0,57	0,39	0,79	0,16	0,31	0,87	0,56	0,47	0,39	1,00
**Antiplatelet drugs**	**0,02**	**0,00**	0,16	**0,05**	**0,00**	**0,00**	**0,01**	**0,00**	0,06	**0,02**	**0,00**		**0,00**	**0,00**	1,00	**0,02**	0,17	0,24	**0,01**	0,78	0,89	0,99	0,11	0,43	0,19	0,50	1,00	0,82
**Anticoagulants usage**	0,50	**0,00**	0,79	**0,00**	1,00	0,42	0,25	0,73	0,23	0,61	0,42	**0,00**		0,62	0,14	0,93	0,99	**0,00**	**0,00**	0,71	1,00	0,11	0,69	**0,02**	0,74	0,75	0,48	**0,00**
**Direct oral anticoagulants usage**	0,17	**0,01**	0,11	**0,00**	1,00	0,09	0,75	**0,01**	1,00	0,57	0,63	**0,00**	0,62		1,00	0,10	0,57	**0,00**	**0,00**	**0,02**	0,22	0,12	0,34	0,35	0,87	0,79	0,57	0,89
**Wake-up stroke**	**0,04**	0,57	0,51	0,68	0,44	0,20	0,53	0,39	0,69	0,52	0,10	1,00	0,14	1,00		0,49	**0,00**	0,81	**0,00**	0,51	0,45	**0,00**	0,31	0,36	0,63	0,33	1,00	0,43
**Pre mRS**	1,00	**0,00**	**0,00**	**0,01**	**0,00**	0,12	0,48	**0,03**	0,13	0,12	0,59	**0,02**	0,93	0,10	0,49		0,94	**0,02**	**0,00**	0,37	1,00	**0,00**	0,93	0,76	0,80	0,41	1,00	0,46
**ASPECTS**	0,36	0,08	0,35	0,83	1,00	0,64	0,44	0,71	0,85	0,61	0,35	0,17	0,99	0,57	**0,00**	0,94		0,64	**0,00**	0,75	0,92	**0,01**	0,60	0,13	0,91	1,00	0,09	0,28
**TOAST**	**0,00**	0,07	0,66	**0,00**	0,62	**0,00**	**0,00**	**0,01**	**0,04**	**0,00**	0,89	0,24	**0,00**	**0,00**	0,81	**0,02**	0,64		**0,00**	**0,00**	0,37	0,05	0,24	0,31	0,36	0,72	0,85	0,96
**Thrombolysis**	0,13	**0,00**	0,07	**0,00**	**0,03**	0,71	1,00	0,10	0,39	0,75	0,57	**0,01**	**0,00**	**0,00**	**0,00**	**0,00**	**0,00**	**0,00**		0,66	0,16	0,64	0,22	0,25	0,26	0,29	1,00	0,16
**Vessel type**	0,19	0,53	0,49	0,07	**0,02**	0,47	0,15	0,86	0,19	0,17	0,39	0,78	0,71	**0,02**	0,51	0,37	0,75	**0,00**	0,66		0,68	**0,00**	0,16	0,29	0,08	0,75	0,85	0,60
**Vessel side**	0,21	0,52	0,96	0,45	0,40	0,19	**0,00**	0,72	0,14	0,07	0,79	0,89	1,00	0,22	0,45	1,00	0,92	0,37	0,16	0,68		0,42	0,30	0,65	0,32	0,75	1,00	0,57
**Leptomeningeal collaterals**	0,58	0,25	0,30	**0,02**	0,38	0,36	0,93	0,39	0,71	0,44	0,16	0,99	0,11	0,12	**0,00**	**0,00**	**0,01**	0,05	0,64	**0,00**	0,42		0,79	0,48	0,76	0,57	0,08	0,26
**Procedure type**	0,68	0,71	0,95	0,24	**0,04**	**0,01**	0,50	0,88	0,17	0,08	0,31	0,11	0,69	0,34	0,31	0,93	0,60	0,24	0,22	0,16	0,30	0,79		0,51	0,42	0,71	0,74	0,53
**Procedure complications**	0,43	0,29	0,62	0,34	0,27	0,39	0,30	0,45	0,40	0,41	0,87	0,43	**0,02**	0,35	0,36	0,76	0,13	0,31	0,25	0,29	0,65	0,48	0,51		**0,04**	0,77	0,56	0,28
**Vessel perforation**	0,29	0,51	0,69	0,69	0,33	1,00	**0,05**	0,61	0,29	0,14	0,56	0,19	0,74	0,87	0,63	0,80	0,91	0,36	0,26	0,08	0,32	0,76	0,42	**0,04**		0,15	0,18	0,92

**TICI**	1,00	0,74	0,86	0,77	0,50	1,00	0,54	0,19	0,81	0,34	0,47	0,50	0,75	0,79	0,33	0,41	1,00	0,72	0,29	0,75	0,75	0,57	0,71	0,77	0,15		1,00	0,61
**ICA stenting**	0,82	0,43	**0,02**	0,64	1,00	0,99	0,70	0,68	0,25	1,00	0,39	1,00	0,48	0,57	1,00	1,00	0,09	0,85	1,00	0,85	1,00	0,08	0,74	0,56	0,18	1,00		0,30
**Bleeding class**	0,13	0,09	0,64	0,56	0,23	0,87	0,51	0,52	0,41	0,51	1,00	0,82	**0,00**	0,89	0,43	0,46	0,28	0,96	0,16	0,60	0,57	0,26	0,53	0,28	0,92	0,61	0,30	

Supplementary table S3. Continuous features correlation.

	Age	CRP	Glycemia	Creatinine	Cholesterol	Triglycerides	High density lipoprotein (HDL)	Low density lipoprotein (LDL)	White Blood Cells (WBCs)	Neutrophils (Neu)	Lymphocytes (Ly)	Red Blood cells (RBCs)	Hematocrit (HCT)	Platelets (PLT)	Fibrinogen	INR	Neutrophil Lymphocyte ratio (NLR)	Systemic Inflammatory Index (SII)	Baseline NIHSS	Onset to puncture time	Onset to the procedure end time	Puncture to end time	Number of steps
**Age**	1,00	0,05	0,06	0,13	-0,12	-0,18	0,12	-0,14	-0,15	-0,01	-0,05	-0,24	-0,17	0,02	0,10	0,11	0,08	0,09	0,18	0,02	-0,01	-0,02	-0,18
**CRP**	0,05	1,00	0,10	0,02	-0,08	-0,01	-0,16	-0,07	0,21	0,14	-0,17	-0,04	-0,12	0,03	0,37	0,02	0,18	0,11	0,01	0,09	0,08	0,00	0,08
**Glycemia**	0,06	0,10	1,00	0,08	-0,09	0,10	-0,15	-0,09	0,22	0,14	-0,11	0,01	-0,06	0,03	0,21	-0,01	0,12	0,09	0,07	0,11	0,12	-0,01	0,04
**Creatinine**	0,13	0,02	0,08	1,00	-0,10	0,02	-0,09	-0,09	0,02	0,02	-0,07	-0,07	-0,02	0,02	0,16	0,11	-0,02	-0,01	-0,03	-0,04	0,00	0,07	0,00
**Cholesterol**	-0,12	-0,08	-0,09	-0,10	1,00	0,33	0,35	0,93	0,05	-0,06	0,10	0,16	0,17	0,00	-0,02	-0,14	-0,09	-0,11	-0,01	0,00	0,01	-0,08	-0,04
**Triglycerides**	-0,18	-0,01	0,10	0,02	0,33	1,00	-0,33	0,29	0,18	-0,07	0,10	0,18	0,18	0,09	0,11	-0,05	-0,03	0,07	-0,04	0,00	0,04	0,02	0,04
**High density lipoprotein (HDL)**	0,12	-0,16	-0,15	-0,09	0,35	-0,33	1,00	0,12	-0,15	-0,01	0,01	-0,07	-0,02	-0,12	-0,15	-0,04	-0,01	-0,06	0,07	0,00	-0,03	-0,05	-0,12
**Low density lipoprotein (LDL)**	-0,14	-0,07	-0,09	-0,09	0,93	0,29	0,12	1,00	0,07	-0,06	0,10	0,18	0,17	0,03	-0,01	-0,14	-0,09	-0,10	-0,05	-0,01	0,01	-0,07	0,00
**White Blood Cells (WBCs)**	-0,15	0,21	0,22	0,02	0,05	0,18	-0,15	0,07	1,00	0,41	-0,35	0,10	0,09	0,28	0,16	-0,06	0,41	0,42	0,05	0,15	0,13	-0,02	0,02
**Neutrophils (Neu)**	-0,01	0,14	0,14	0,02	-0,06	-0,07	-0,01	-0,06	0,41	1,00	-0,95	-0,18	-0,10	0,07	0,07	0,05	0,75	0,54	0,06	0,23	0,19	0,06	0,00
**Lymphocytes (Ly)**	-0,05	-0,17	-0,11	-0,07	0,10	0,10	0,01	0,10	-0,35	-0,95	1,00	0,21	0,11	-0,08	-0,09	-0,06	-0,73	-0,54	-0,06	-0,21	-0,17	-0,08	-0,01
**Red Blood cells (RBCs)**	-0,24	-0,04	0,01	-0,07	0,16	0,18	-0,07	0,18	0,10	-0,18	0,21	1,00	0,49	0,05	0,01	-0,06	-0,04	0,01	-0,14	0,07	0,06	-0,01	0,06
**Hematocrit (HCT)**	-0,17	-0,12	-0,06	-0,02	0,17	0,18	-0,02	0,17	0,09	-0,10	0,11	0,49	1,00	-0,08	-0,04	-0,05	-0,08	-0,05	-0,06	-0,02	-0,06	0,05	-0,02
**Platelets (PLT)**	0,02	0,03	0,03	0,02	0,00	0,09	-0,12	0,03	0,28	0,07	-0,08	0,05	-0,08	1,00	0,16	-0,01	0,10	0,61	-0,02	0,03	0,04	-0,02	-0,04
**Fibrinogen**	0,10	0,37	0,21	0,16	-0,02	0,11	-0,15	-0,01	0,16	0,07	-0,09	0,01	-0,04	0,16	1,00	0,05	0,06	0,08	0,02	0,10	0,12	0,06	0,05
**INR**	0,11	0,02	-0,01	0,11	-0,14	-0,05	-0,04	-0,14	-0,06	0,05	-0,06	-0,06	-0,05	-0,01	0,05	1,00	0,04	0,04	0,09	0,10	0,09	-0,03	-0,05
**Neutrophil Lymphocyte ratio (NLR)**	0,08	0,18	0,12	-0,02	-0,09	-0,03	-0,01	-0,09	0,41	0,75	-0,73	-0,04	-0,08	0,10	0,06	0,04	1,00	0,77	0,08	0,28	0,21	0,02	-0,02
**Systemic Inflammatory Index (SII)**	0,09	0,11	0,09	-0,01	-0,11	0,07	-0,06	-0,10	0,42	0,54	-0,54	0,01	-0,05	0,61	0,08	0,04	0,77	1,00	0,05	0,21	0,17	0,00	-0,05

**Baseline NIHSS**	0,18	0,01	0,07	-0,03	-0,01	-0,04	0,07	-0,05	0,05	0,06	-0,06	-0,14	-0,06	-0,02	0,02	0,09	0,08	0,05	1,00	-0,06	-0,08	0,01	0,07
**Onset to puncture time**	0,02	0,09	0,11	-0,04	0,00	0,00	0,00	-0,01	0,15	0,23	-0,21	0,07	-0,02	0,03	0,10	0,10	0,28	0,21	-0,06	1,00	0,89	-0,01	0,01
**Onset to the procedure end time**	-0,01	0,08	0,12	0,00	0,01	0,04	-0,03	0,01	0,13	0,19	-0,17	0,06	-0,06	0,04	0,12	0,09	0,21	0,17	-0,08	0,89	1,00	0,16	0,12
**Puncture to end time**	-0,02	0,00	-0,01	0,07	-0,08	0,02	-0,05	-0,07	-0,02	0,06	-0,08	-0,01	0,05	-0,02	0,06	-0,03	0,02	0,00	0,01	-0,01	0,16	1,00	0,52
**Number of steps**	-0,18	0,08	0,04	0,00	-0,04	0,04	-0,12	0,00	0,02	0,00	-0,01	0,06	-0,02	-0,04	0,05	-0,05	-0,02	-0,05	0,07	0,01	0,12	0,52	1,00

Supplementary table S4. Cut-off values for continuous features.

Cutpoint	Sensitivity (%)	Specificity (%)	Positive predictive value (%)	Negative predictive value (%)	Youden's index	Area Under the Curve	Metric Score
**Age**							
67.44	90.23%	33.48%	27.78%	92.35%	0.24	0.68	1.24
68	90.23%	33.69%	27.84%	92.40%	0.24	0.68	1.24
68.14	89.47%	34.75%	28%	92.09%	0.24	0.68	1.24
68.33	89.47%	34.97%	28.07%	92.13%	0.24	0.68	1.24
69	88.72%	34.97%	27.90%	91.62%	0.24	0.68	1.24
69.52	87.22%	36.25%	27.95%	90.91%	0.24	0.68	1.23
69.65	87.22%	36.46%	28.02%	90.96%	0.24	0.68	1.24
70.11	84.96%	38.81%	28.25%	90.10%	0.24	0.68	1.24
70.31	84.96%	39.02%	28.32%	90.15%	0.24	0.68	1.24
70.53	84.96%	39.23%	28.39%	90.20%	0.24	0.68	1.24
71	84.96%	39.45%	28.46%	90.24%	0.24	0.68	1.24
71.8	81.95%	41.36%	28.39%	88.99%	0.23	0.68	1.23
72.37	79.70%	43.71%	28.65%	88.36%	0.23	0.68	1.23
72.51	79.70%	43.92%	28.73%	88.41%	0.24	0.68	1.24
72.59	79.70%	44.14%	28.80%	88.46%	0.24	0.68	1.24
72.91	79.70%	44.35%	28.88%	88.51%	0.24	0.68	1.24
73	79.70%	44.56%	28.96%	88.56%	0.24	0.68	1.24
73.06	78.20%	46.91%	29.46%	88.35%	0.25	0.68	1.25
73.17	78.20%	47.12%	29.55%	88.40%	0.25	0.68	1.25
73.24	78.20%	47.33%	29.63%	88.45%	0.26	0.68	1.26
73.29	78.20%	47.55%	29.71%	88.49%	0.26	0.68	1.26
73.31	77.44%	47.55%	29.51%	88.14%	0.25	0.68	1.25
73.5	77.44%	47.76%	29.60%	88.19%	0.25	0.68	1.25
73.73	77.44%	47.97%	29.68%	88.24%	0.25	0.68	1.25
74	77.44%	48.19%	29.77%	88.28%	0.26	0.68	1.26
74.18	74.44%	50.75%	30%	87.50%	0.25	0.68	1.25
74.49	74.44%	50.96%	30.09%	87.55%	0.25	0.68	1.25
74.63	74.44%	51.17%	30.18%	87.59%	0.26	0.68	1.26

74.84	74.44%	51.39%	30.28%	87.64%	0.26	0.68	1.26
74.92	74.44%	51.60%	30.37%	87.68%	0.26	0.68	1.26
75	73.68%	51.60%	30.15%	87.36%	0.25	0.68	1.25
75.23	69.92%	53.52%	29.90%	86.25%	0.23	0.68	1.23
75.34	69.92%	53.73%	30%	86.30%	0.24	0.68	1.24
75.45	69.92%	53.94%	30.10%	86.35%	0.24	0.68	1.24
75.89	69.17%	54.16%	29.97%	86.10%	0.23	0.68	1.23
76	69.17%	54.37%	30.07%	86.15%	0.24	0.68	1.24
76.03	66.17%	57.36%	30.56%	85.67%	0.24	0.68	1.24
76.14	66.17%	57.57%	30.66%	85.71%	0.24	0.68	1.24
76.21	66.17%	57.78%	30.77%	85.76%	0.24	0.68	1.24
76.96	66.17%	58.21%	30.99%	85.85%	0.24	0.68	1.24
77	66.17%	58.42%	31.10%	85.89%	0.25	0.68	1.25
77.1	64.66%	61.41%	32.21%	85.97%	0.26	0.68	1.26
77.21	64.66%	61.62%	32.33%	86.01%	0.26	0.68	1.26
77.37	64.66%	61.83%	32.45%	86.05%	0.27	0.68	1.26
77.79	64.66%	62.05%	32.58%	86.09%	0.27	0.68	1.27
77.92	64.66%	62.47%	32.82%	86.18%	0.27	0.68	1.27
78	64.66%	62.69%	32.95%	86.22%	0.27	0.68	1.27
78.1	62.41%	65.67%	34.02%	86.03%	0.28	0.68	1.28
78.57	62.41%	65.88%	34.16%	86.07%	0.28	0.68	1.28
78.66	61.65%	65.88%	33.88%	85.83%	0.28	0.68	1.28
78.79	61.65%	66.10%	34.02%	85.87%	0.28	0.68	1.28
78.85	61.65%	66.31%	34.17%	85.91%	0.28	0.68	1.28
79	61.65%	66.52%	34.31%	85.95%	0.28	0.68	1.28
79.1	57.14%	67.59%	33.33%	84.76%	0.25	0.68	1.25
79.29	56.39%	67.59%	33.04%	84.53%	0.24	0.68	1.24
79.51	55.64%	67.80%	32.89%	84.35%	0.23	0.68	1.23
79.73	55.64%	68.02%	33.04%	84.39%	0.24	0.68	1.24
80.69	52.63%	70.79%	33.82%	84.05%	0.23	0.68	1.23
**Glycemia**							
102	90.98%	17.70%	23.87%	87.37%	0.09	0.59	01.09
103	89.47%	19.83%	24.04%	86.92%	0.09	0.59	01.09
104	88.72%	21.32%	24.23%	86.96%	0.10	0.59	1.10

105	86.47%	23.03%	24.16%	85.71%	0.09	0.59	01.09
106	84.96%	24.52%	24.20%	85.19%	0.09	0.59	01.09
107	83.46%	26.44%	24.34%	84.93%	0.10	0.59	1.10
108	81.20%	28.36%	24.32%	84.18%	0.10	0.59	1.10
109	79.70%	30.49%	24.54%	84.12%	0.10	0.59	1.10
110	77.44%	33.90%	24.94%	84.13%	0.11	0.59	1.11
111	77.44%	34.75%	25.18%	84.46%	0.12	0.59	1.12
112	75.94%	37.10%	25.51%	84.47%	0.13	0.59	1.13
113	73.68%	37.95%	25.19%	83.57%	0.12	0.59	1.12
114	72.93%	39.45%	25.46%	83.71%	0.12	0.59	1.12
115	72.18%	41.15%	25.81%	83.91%	0.13	0.59	1.13
116	69.17%	43.07%	25.63%	83.13%	0.12	0.59	1.12
117	67.67%	44.14%	25.57%	82.80%	0.12	0.59	1.12
118	64.66%	45.84%	25.29%	82.06%	0.11	0.59	1.11
119	63.91%	48.83%	26.15%	82.67%	0.13	0.59	1.13
120	63.16%	50.11%	26.42%	82.75%	0.13	0.59	1.13
121	56.39%	54.37%	25.95%	81.47%	0.11	0.59	1.11
122	55.64%	56.08%	26.43%	81.68%	0.12	0.59	1.12
123	52.63%	57.36%	25.93%	81.02%	0.10	0.59	1.10
124	50.38%	58.42%	25.57%	80.59%	0.09	0.59	01.09
125	48.87%	59.91%	25.69%	80.52%	0.09	0.59	01.09
126	48.87%	60.34%	25.90%	80.63%	0.09	0.59	01.09
127	47.37%	62.26%	26.25%	80.66%	0.10	0.59	1.10
128	46.62%	63.54%	26.61%	80.76%	0.10	0.59	1.10
129	45.11%	64.61%	26.55%	80.59%	0.10	0.59	1.10
130	43.61%	65.03%	26.13%	80.26%	0.09	0.59	01.09
132	40.60%	68.23%	26.60%	80.20%	0.09	0.59	01.09
134	39.10%	70.15%	27.08%	80.24%	0.09	0.59	01.09
135	38.35%	71.22%	27.42%	80.29%	0.10	0.59	1.10
136	38.35%	72.07%	28.02%	80.48%	0.10	0.59	1.10
137	38.35%	72.71%	28.49%	80.61%	0.11	0.59	1.11
138	36.84%	73.56%	28.32%	80.42%	0.10	0.59	1.10
139	35.34%	73.77%	27.65%	80.09%	0.09	0.59	01.09
141	33.83%	74.63%	27.44%	79.91%	0.08	0.59	01.08
142	33.83%	75.69%	28.30%	80.14%	0.10	0.59	1.10

143	33.83%	76.76%	29.22%	80.36%	0.11	0.59	1.11
144	33.08%	78.04%	29.93%	80.44%	0.11	0.59	1.11
145	32.33%	78.68%	30.07%	80.39%	0.11	0.59	1.11
146	30.83%	79.53%	29.93%	80.22%	0.10	0.59	1.10
147	30.08%	79.74%	29.63%	80.09%	0.10	0.59	1.10
148	30.08%	80.81%	30.77%	80.30%	0.11	0.59	1.11
149	30.08%	81.02%	31.01%	80.34%	0.11	0.59	1.11
150	29.32%	81.66%	31.20%	80.29%	0.11	0.59	1.11
151	29.32%	81.88%	31.45%	80.33%	0.11	0.59	1.11
152	28.57%	83.37%	32.76%	80.45%	0.12	0.59	1.12
154	26.32%	84.01%	31.82%	80.08%	0.10	0.59	1.10
155	25.56%	84.86%	32.38%	80.08%	0.10	0.59	1.10
156	25.56%	85.07%	32.69%	80.12%	0.11	0.59	1.11
157	25.56%	85.29%	33.01%	80.16%	0.11	0.59	1.11
158	24.81%	85.29%	32.35%	80%	0.10	0.59	1.10
159	24.81%	85.71%	33%	80.08%	0.11	0.59	1.11
160	24.81%	86.14%	33.67%	80.16%	0.11	0.59	1.11
161	23.31%	86.35%	32.63%	79.88%	0.10	0.59	1.10
162	22.56%	86.35%	31.91%	79.72%	0.09	0.59	01.09
163	22.56%	86.78%	32.61%	79.80%	0.09	0.59	01.09
164	22.56%	87.85%	34.48%	80%	0.10	0.59	1.10
165	22.56%	88.06%	34.88%	80.04%	0.11	0.59	1.11
169	21.05%	88.27%	33.73%	79.77%	0.09	0.59	01.09
170	21.05%	88.91%	35%	79.89%	0.10	0.59	1.10
171	20.30%	89.13%	34.62%	79.77%	0.09	0.59	01.09
172	20.30%	89.34%	35.06%	79.81%	0.10	0.59	1.10
173	20.30%	89.77%	36%	79.89%	0.10	0.59	1.10
174	18.80%	89.98%	34.72%	79.62%	0.09	0.59	01.09
177	17.29%	91.04%	35.38%	79.52%	0.08	0.59	01.08
178	17.29%	91.26%	35.94%	79.55%	0.09	0.59	01.09
204	12.03%	96.38%	48.48%	79.44%	0.08	0.59	01.08
**Neutrophil-to-lymphocyte ratio**							
2.13	87.97%	21.96%	24.22%	86.55%	0.10	0.59	1.10
2.14	87.97%	22.17%	24.27%	86.67%	0.10	0.59	1.10

2.14	87.22%	22.60%	24.22%	86.18%	0.10	0.59	1.10
2.16	87.22%	23.03%	24.32%	86.40%	0.10	0.59	1.10
2.18	87.22%	23.24%	24.37%	86.51%	0.10	0.59	1.10
2.21	86.47%	23.24%	24.21%	85.83%	0.10	0.59	1.10
2.22	86.47%	24.09%	24.42%	86.26%	0.11	0.59	1.11
2.25	86.47%	24.31%	24.47%	86.36%	0.11	0.59	1.11
2.29	86.47%	24.95%	24.63%	86.67%	0.11	0.59	1.11
2.30	86.47%	25.16%	24.68%	86.76%	0.12	0.59	1.12
2.31	86.47%	25.59%	24.78%	86.96%	0.12	0.59	1.12
2.32	85.71%	25.80%	24.68%	86.43%	0.12	0.59	1.12
2.33	85.71%	26.01%	24.73%	86.52%	0.12	0.59	1.12
2.35	84.96%	26.44%	24.67%	86.11%	0.11	0.59	1.11
2.36	84.96%	26.65%	24.73%	86.21%	0.12	0.59	1.12
2.37	84.96%	26.87%	24.78%	86.30%	0.12	0.59	1.12
2.38	83.46%	27.08%	24.50%	85.23%	0.11	0.59	1.11
2.41	83.46%	27.29%	24.56%	85.33%	0.11	0.59	1.11
2.42	83.46%	27.51%	24.61%	85.43%	0.11	0.59	1.11
2.44	83.46%	27.72%	24.67%	85.53%	0.11	0.59	1.11
2.44	82.71%	27.93%	24.55%	85.06%	0.11	0.59	1.11
2.46	82.71%	28.14%	24.61%	85.16%	0.11	0.59	1.11
2.48	82.71%	28.78%	24.77%	85.44%	0.11	0.59	1.11
2.50	82.71%	29.21%	24.89%	85.62%	0.12	0.59	1.12
2.52	81.95%	29.21%	24.72%	85.09%	0.11	0.59	1.11
2.54	81.95%	29.42%	24.77%	85.19%	0.11	0.59	1.11
2.54	81.95%	29.64%	24.83%	85.28%	0.12	0.59	1.12
2.56	81.95%	29.85%	24.89%	85.37%	0.12	0.59	1.12
2.60	81.95%	30.49%	25.06%	85.63%	0.12	0.59	1.12
2.63	81.20%	30.92%	25%	85.29%	0.12	0.59	1.12
2.64	81.20%	31.13%	25.06%	85.38%	0.12	0.59	1.12
2.67	80.45%	31.77%	25.06%	85.14%	0.12	0.59	1.12
2.68	79.70%	31.98%	24.94%	84.75%	0.12	0.59	1.12
2.70	79.70%	32.20%	25%	84.83%	0.12	0.59	1.12
2.71	79.70%	32.41%	25.06%	84.92%	0.12	0.59	1.12
2.72	79.70%	32.84%	25.18%	85.08%	0.13	0.59	1.13
2.73	79.70%	33.05%	25.24%	85.16%	0.13	0.59	1.13

2.75	78.95%	33.05%	25.06%	84.70%	0.12	0.59	1.12
2.77	78.95%	33.48%	25.18%	84.86%	0.12	0.59	1.12
2.78	78.20%	33.48%	25%	84.41%	0.12	0.59	1.12
2.79	78.20%	33.90%	25.12%	84.57%	0.12	0.59	1.12
2.80	76.69%	33.90%	24.76%	83.68%	0.11	0.59	1.11
2.82	75.94%	33.90%	24.57%	83.25%	0.10	0.59	1.10
2.83	75.94%	34.12%	24.63%	83.33%	0.10	0.59	1.10
2.83	75.94%	34.54%	24.75%	83.51%	0.10	0.59	1.10
2.86	75.19%	34.54%	24.57%	83.08%	0.10	0.59	1.10
2.92	74.44%	35.39%	24.63%	83%	0.10	0.59	1.10
3.04	73.68%	36.03%	24.62%	82.84%	0.10	0.59	1.10
3.05	73.68%	36.46%	24.75%	83.01%	0.10	0.59	1.10
3.05	73.68%	36.89%	24.87%	83.17%	0.11	0.59	1.11
3.09	73.68%	37.10%	24.94%	83.25%	0.11	0.59	1.11
3.14	72.93%	37.10%	24.74%	82.86%	0.10	0.59	1.10
3.18	72.93%	38.38%	25.13%	83.33%	0.11	0.59	1.11
3.19	72.18%	38.81%	25.07%	83.11%	0.11	0.59	1.11
3.23	70.68%	38.81%	24.67%	82.35%	0.09	0.59	01.09
3.23	70.68%	39.02%	24.74%	82.43%	0.10	0.59	1.10
3.24	70.68%	39.23%	24.80%	82.51%	0.10	0.59	1.10
3.27	70.68%	39.45%	24.87%	82.59%	0.10	0.59	1.10
3.30	70.68%	39.87%	25%	82.74%	0.11	0.59	1.11
3.32	69.92%	39.87%	24.80%	82.38%	0.10	0.59	1.10
3.35	69.92%	40.09%	24.87%	82.46%	0.10	0.59	1.10
3.36	69.92%	40.30%	24.93%	82.53%	0.10	0.59	1.10
3.38	69.92%	40.51%	25%	82.61%	0.10	0.59	1.10
3.40	69.17%	40.51%	24.80%	82.25%	0.10	0.59	1.10
3.43	69.17%	40.72%	24.86%	82.33%	0.10	0.59	1.10
3.44	69.17%	41.15%	25%	82.48%	0.10	0.59	1.10
3.45	69.17%	41.36%	25.07%	82.55%	0.11	0.59	1.11
3.50	69.17%	41.58%	25.14%	82.63%	0.11	0.59	1.11
3.55	68.42%	42%	25.07%	82.43%	0.10	0.59	1.10
3.58	68.42%	42.22%	25.14%	82.50%	0.11	0.59	1.11
3.60	68.42%	42.43%	25.21%	82.57%	0.11	0.59	1.11
3.61	68.42%	43.07%	25.42%	82.79%	0.11	0.59	1.11

3.65	68.42%	43.28%	25.49%	82.86%	0.12	0.59	1.12
3.68	68.42%	44.14%	25.78%	83.13%	0.13	0.59	1.13
3.70	68.42%	44.56%	25.93%	83.27%	0.13	0.59	1.13
3.73	68.42%	44.78%	26%	83.33%	0.13	0.59	1.13
3.74	68.42%	44.99%	26.07%	83.40%	0.13	0.59	1.13
3.79	68.42%	45.42%	26.22%	83.53%	0.14	0.59	1.14
3.80	68.42%	46.06%	26.45%	83.72%	0.14	0.59	1.14
3.84	67.67%	46.06%	26.24%	83.40%	0.14	0.59	1.14
3.87	67.67%	46.27%	26.32%	83.46%	0.14	0.59	1.14
3.89	66.92%	46.27%	26.10%	83.14%	0.13	0.59	1.13
3.89	66.92%	46.70%	26.25%	83.27%	0.14	0.59	1.14
3.90	66.17%	47.12%	26.19%	83.08%	0.13	0.59	1.13
3.94	65.41%	47.12%	25.97%	82.77%	0.13	0.59	1.13
3.95	65.41%	47.33%	26.05%	82.84%	0.13	0.59	1.13
4.87	43.61%	66.74%	27.10%	80.67%	0.10	0.59	1.10
4.88	42.86%	66.74%	26.76%	80.46%	0.10	0.59	1.10
4.93	42.11%	68.02%	27.18%	80.56%	0.10	0.59	1.10
4.94	42.11%	68.23%	27.32%	80.60%	0.10	0.59	1.10
5.00	42.11%	68.66%	27.59%	80.70%	0.11	0.59	1.11
5.07	41.35%	68.66%	27.23%	80.50%	0.10	0.59	1.10
5.13	41.35%	69.08%	27.50%	80.60%	0.10	0.59	1.10
5.20	40.60%	69.51%	27.41%	80.49%	0.10	0.59	1.10
5.21	39.85%	70.15%	27.46%	80.44%	0.10	0.59	1.10
5.27	39.85%	70.36%	27.60%	80.49%	0.10	0.59	1.10
5.29	39.85%	70.79%	27.89%	80.58%	0.11	0.59	1.11
5.33	39.85%	71.22%	28.19%	80.68%	0.11	0.59	1.11
5.40	39.10%	71.64%	28.11%	80.58%	0.11	0.59	1.11
5.43	39.10%	71.86%	28.26%	80.62%	0.11	0.59	1.11
5.47	38.35%	72.07%	28.02%	80.48%	0.10	0.59	1.10
5.54	38.35%	72.28%	28.18%	80.52%	0.11	0.59	1.11
5.57	38.35%	72.71%	28.49%	80.61%	0.11	0.59	1.11
5.71	37.59%	73.56%	28.74%	80.61%	0.11	0.59	1.11
5.79	36.84%	73.77%	28.49%	80.47%	0.11	0.59	1.11
5.85	36.84%	74.20%	28.82%	80.56%	0.11	0.59	1.11
5.86	36.84%	74.63%	29.17%	80.65%	0.11	0.59	1.11

5.92	36.09%	75.05%	29.09%	80.55%	0.11	0.59	1.11
5.92	36.09%	75.27%	29.27%	80.59%	0.11	0.59	1.11
5.93	36.09%	75.48%	29.45%	80.64%	0.12	0.59	1.12
6.00	36.09%	75.69%	29.63%	80.68%	0.12	0.59	1.12
6.15	35.34%	76.12%	29.56%	80.59%	0.11	0.59	1.11
6.23	35.34%	76.33%	29.75%	80.63%	0.12	0.59	1.12
6.31	34.59%	76.33%	29.30%	80.45%	0.11	0.59	1.11
6.42	34.59%	76.97%	29.87%	80.58%	0.12	0.59	1.12
6.50	34.59%	77.40%	30.26%	80.67%	0.12	0.59	1.12
6.58	33.83%	77.83%	30.20%	80.57%	0.12	0.59	1.12
6.67	33.83%	78.68%	31.03%	80.74%	0.13	0.59	1.13
6.75	33.08%	79.10%	30.99%	80.65%	0.12	0.59	1.12
6.83	32.33%	79.10%	30.50%	80.48%	0.11	0.59	1.11
6.92	32.33%	79.32%	30.71%	80.52%	0.12	0.59	1.12
7.00	31.58%	79.74%	30.66%	80.43%	0.11	0.59	1.11
7.08	30.83%	80.60%	31.06%	80.43%	0.11	0.59	1.11
7.09	30.83%	80.81%	31.30%	80.47%	0.12	0.59	1.12
7.18	30.83%	81.02%	31.54%	80.51%	0.12	0.59	1.12
7.36	30.08%	81.24%	31.25%	80.38%	0.11	0.59	1.11
7.45	29.32%	81.45%	30.95%	80.25%	0.11	0.59	1.11
7.55	29.32%	81.88%	31.45%	80.33%	0.11	0.59	1.11
7.56	29.32%	82.30%	31.97%	80.42%	0.12	0.59	1.12
7.70	29.32%	82.52%	32.23%	80.46%	0.12	0.59	1.12
7.82	28.57%	82.52%	31.67%	80.29%	0.11	0.59	1.11
7.90	27.07%	82.52%	30.51%	79.96%	0.10	0.59	1.10
8.00	27.07%	82.94%	31.03%	80.04%	0.10	0.59	1.10
8.10	27.07%	83.37%	31.58%	80.12%	0.10	0.59	1.10
8.20	27.07%	83.58%	31.86%	80.16%	0.11	0.59	1.11
8.30	26.32%	84.01%	31.82%	80.08%	0.10	0.59	1.10
8.40	26.32%	84.65%	32.71%	80.20%	0.11	0.59	1.11
8.50	26.32%	86.14%	35%	80.48%	0.12	0.59	1.12
8.60	26.32%	86.99%	36.46%	80.63%	0.13	0.59	1.13
8.67	25.56%	87.42%	36.56%	80.55%	0.13	0.59	1.13
8.70	24.81%	87.63%	36.26%	80.43%	0.12	0.59	1.12
9.11	24.81%	87.85%	36.67%	80.47%	0.13	0.59	1.13

9.33	24.81%	88.06%	37.08%	80.51%	0.13	0.59	1.13
9.44	24.81%	88.70%	38.37%	80.62%	0.14	0.59	1.14
9.50	24.81%	89.13%	39.29%	80.69%	0.14	0.59	1.14
9.56	24.06%	89.13%	38.55%	80.54%	0.13	0.59	1.13
9.67	24.06%	89.34%	39.02%	80.58%	0.13	0.59	1.13
9.75	23.31%	89.55%	38.75%	80.46%	0.13	0.59	1.13
10.00	22.56%	89.55%	37.97%	80.31%	0.12	0.59	1.12
10.13	21.80%	89.77%	37.66%	80.19%	0.12	0.59	1.12
10.38	21.05%	89.77%	36.84%	80.04%	0.11	0.59	1.11
10.50	21.05%	89.98%	37.33%	80.08%	0.11	0.59	1.11
10.63	19.55%	89.98%	35.62%	79.77%	0.10	0.59	1.10
10.75	19.55%	90.41%	36.62%	79.85%	0.10	0.59	1.10
**International normalized ratio (INR)**							
1.21	25.56%	87.85%	37.36%	80.63%	0.13	0.58	1.13
1.22	24.06%	88.49%	37.21%	80.43%	0.13	0.58	1.13
1.23	23.31%	89.13%	37.80%	80.38%	0.12	0.58	1.12
1.24	22.56%	89.98%	38.96%	80.38%	0.13	0.58	1.13
1.25	21.80%	90.62%	39.73%	80.34%	0.12	0.58	1.12
1.26	21.05%	90.83%	39.44%	80.23%	0.12	0.58	1.12
1.27	21.05%	91.04%	40%	80.26%	0.12	0.58	1.12
1.29	21.05%	91.47%	41.18%	80.34%	0.13	0.58	1.13
1.30	20.30%	91.47%	40.30%	80.19%	0.12	0.58	1.12
1.31	18.80%	91.68%	39.06%	79.93%	0.10	0.58	1.10
1.32	18.80%	91.90%	39.68%	79.96%	0.11	0.58	1.11
1.33	18.05%	91.90%	38.71%	79.81%	0.10	0.58	1.10
1.35	18.05%	92.11%	39.34%	79.85%	0.10	0.58	1.10
1.36	18.05%	92.32%	40%	79.89%	0.10	0.58	1.10
1.37	18.05%	92.75%	41.38%	79.96%	0.11	0.58	1.11
1.38	17.29%	92.75%	40.35%	79.82%	0.10	0.58	1.10
1.39	16.54%	92.75%	39.29%	79.67%	0.09	0.58	01.09
1.42	15.04%	93.60%	40%	79.53%	0.09	0.58	01.09
**Baseline NIHSS value**							
15	81.95%	35.82%	26.59%	87.50%	0.18	0.65	1.18
16	78.95%	40.94%	27.49%	87.27%	0.20	0.65	1.20

17	72.18%	46.70%	27.75%	85.55%	0.19	0.65	1.19
18	66.17%	51.60%	27.94%	84.32%	0.18	0.65	1.18
19	61.65%	60.77%	30.83%	84.82%	0.22	0.65	1.22
20	57.14%	65.46%	31.93%	84.34%	0.23	0.65	1.23
21	45.86%	74.63%	33.89%	82.94%	0.21	0.65	1.20
22	40.60%	77.61%	33.96%	82.17%	0.18	0.65	1.18
**Onset-to-puncture time (OPT)**							
195	42.86%	63.33%	24.89%	79.62%	0.06	0.53	01.06
195	42.11%	63.75%	24.78%	79.52%	0.06	0.53	01.06
198	42.11%	64.18%	25%	79.63%	0.06	0.53	01.06
199	42.11%	64.39%	25.11%	79.68%	0.07	0.53	01.06
200	41.35%	64.39%	24.77%	79.47%	0.06	0.53	01.06
202	40.60%	64.82%	24.66%	79.37%	0.05	0.53	01.05
207	40.60%	65.03%	24.77%	79.43%	0.06	0.53	01.06
208	39.85%	65.03%	24.42%	79.22%	0.05	0.53	01.05
209	39.10%	65.25%	24.19%	79.07%	0.04	0.53	01.04
210	39.10%	65.46%	24.30%	79.12%	0.05	0.53	01.05
215	37.59%	66.74%	24.27%	79.04%	0.04	0.53	01.04
217	37.59%	67.16%	24.51%	79.15%	0.05	0.53	01.05
219	37.59%	67.38%	24.63%	79.20%	0.05	0.53	01.05
220	37.59%	67.59%	24.75%	79.25%	0.05	0.53	01.05
225	36.09%	68.87%	24.74%	79.17%	0.05	0.53	01.05
225	36.09%	69.08%	24.87%	79.22%	0.05	0.53	01.05
228	36.09%	69.30%	25%	79.27%	0.05	0.53	01.05
230	36.09%	69.51%	25.13%	79.32%	0.06	0.53	01.06
231	36.09%	70.15%	25.53%	79.47%	0.06	0.53	01.06
232	36.09%	70.58%	25.81%	79.57%	0.07	0.53	01.07
234	36.09%	70.79%	25.95%	79.62%	0.07	0.53	01.07
235	36.09%	71%	26.09%	79.67%	0.07	0.53	01.07
235	35.34%	71.22%	25.82%	79.52%	0.07	0.53	01.07
237	35.34%	71.43%	25.97%	79.57%	0.07	0.53	01.07
238	35.34%	71.86%	26.26%	79.67%	0.07	0.53	01.07
240	35.34%	72.07%	26.40%	79.72%	0.07	0.53	01.07
240	35.34%	72.28%	26.55%	79.76%	0.08	0.53	01.08

241	34.59%	72.49%	26.29%	79.63%	0.07	0.53	01.07
247	33.83%	72.49%	25.86%	79.44%	0.06	0.53	01.06
248	33.83%	72.71%	26.01%	79.49%	0.07	0.53	01.07
249	33.08%	72.71%	25.58%	79.30%	0.06	0.53	01.06
250	32.33%	72.71%	25.15%	79.12%	0.05	0.53	01.05
252	32.33%	73.77%	25.90%	79.36%	0.06	0.53	01.06
253	31.58%	73.77%	25.45%	79.18%	0.05	0.53	01.05
255	31.58%	73.99%	25.61%	79.22%	0.06	0.53	01.06
258	31.58%	74.20%	25.77%	79.27%	0.06	0.53	01.06
260	31.58%	74.41%	25.93%	79.32%	0.06	0.53	01.06
262	31.58%	75.05%	26.42%	79.46%	0.07	0.53	01.07
264	31.58%	75.27%	26.58%	79.50%	0.07	0.53	01.07
265	31.58%	75.48%	26.75%	79.55%	0.07	0.53	01.07
267	31.58%	75.91%	27.10%	79.64%	0.07	0.53	01.07
270	30.83%	75.91%	26.62%	79.46%	0.07	0.53	01.07
274	30.08%	76.55%	26.67%	79.42%	0.07	0.53	01.07
275	30.08%	76.76%	26.85%	79.47%	0.07	0.53	01.07
276	30.08%	77.19%	27.21%	79.56%	0.07	0.53	01.07
280	30.08%	77.40%	27.40%	79.61%	0.07	0.53	01.07
280	30.08%	78.04%	27.97%	79.74%	0.08	0.53	01.08
282	30.08%	78.25%	28.17%	79.78%	0.08	0.53	01.08
284	30.08%	78.46%	28.37%	79.83%	0.09	0.53	01.09
285	30.08%	78.68%	28.57%	79.87%	0.09	0.53	01.09
286	28.57%	78.68%	27.54%	79.53%	0.07	0.53	01.07
287	28.57%	78.89%	27.74%	79.57%	0.07	0.53	01.07
290	28.57%	79.10%	27.94%	79.61%	0.08	0.53	01.08
292	28.57%	79.32%	28.15%	79.66%	0.08	0.53	01.08
300	27.82%	79.32%	27.61%	79.49%	0.07	0.53	01.07
300	27.07%	79.74%	27.48%	79.41%	0.07	0.53	01.07
303	27.07%	79.96%	27.69%	79.45%	0.07	0.53	01.07
305	27.07%	80.17%	27.91%	79.49%	0.07	0.53	01.07
310	27.07%	80.38%	28.12%	79.54%	0.07	0.53	01.07
310	24.81%	80.81%	26.83%	79.12%	0.06	0.53	01.06
313	24.81%	81.02%	27.05%	79.17%	0.06	0.53	01.06
315	24.81%	81.24%	27.27%	79.21%	0.06	0.53	01.06

320	24.81%	81.45%	27.50%	79.25%	0.06	0.53	01.06
323	24.06%	81.88%	27.35%	79.18%	0.06	0.53	01.06
325	23.31%	81.88%	26.72%	79.01%	0.05	0.53	01.05
330	23.31%	82.30%	27.19%	79.10%	0.06	0.53	01.06
332	23.31%	83.37%	28.44%	79.31%	0.07	0.53	01.07
335	23.31%	83.58%	28.70%	79.35%	0.07	0.53	01.07
337	23.31%	83.80%	28.97%	79.39%	0.07	0.53	01.07
340	23.31%	84.01%	29.25%	79.44%	0.07	0.53	01.07
340	22.56%	84.65%	29.41%	79.40%	0.07	0.53	01.07
345	22.56%	84.86%	29.70%	79.44%	0.07	0.53	01.07
350	22.56%	85.07%	30%	79.48%	0.08	0.53	01.08
351	22.56%	85.29%	30.30%	79.52%	0.08	0.53	01.08
360	22.56%	85.50%	30.61%	79.56%	0.08	0.53	01.08
365	22.56%	85.71%	30.93%	79.60%	0.08	0.53	01.08
365	22.56%	85.93%	31.25%	79.64%	0.08	0.53	01.08
370	22.56%	86.14%	31.58%	79.68%	0.09	0.53	01.09
375	22.56%	86.35%	31.91%	79.72%	0.09	0.53	01.09
380	22.56%	86.57%	32.26%	79.76%	0.09	0.53	01.09
381	21.05%	86.57%	30.77%	79.45%	0.08	0.53	01.08
385	20.30%	86.57%	30%	79.30%	0.07	0.53	01.07
394	19.55%	86.57%	29.21%	79.14%	0.06	0.53	01.06
395	19.55%	86.78%	29.55%	79.18%	0.06	0.53	01.06
400	19.55%	86.99%	29.89%	79.22%	0.07	0.53	01.07
416	19.55%	87.42%	30.59%	79.30%	0.07	0.53	01.07
422	19.55%	87.63%	30.95%	79.34%	0.07	0.53	01.07
430	18.80%	87.63%	30.12%	79.19%	0.06	0.53	01.06
435	18.80%	87.85%	30.49%	79.23%	0.07	0.53	01.07
442	18.05%	87.85%	29.63%	79.08%	0.06	0.53	01.06
460	18.05%	88.06%	30%	79.12%	0.06	0.53	01.06
463	17.29%	88.06%	29.11%	78.97%	0.05	0.53	01.05
470	16.54%	88.06%	28.21%	78.82%	0.05	0.53	01.05
490	16.54%	88.27%	28.57%	78.86%	0.05	0.53	01.05
510	16.54%	88.49%	28.95%	78.90%	0.05	0.53	01.05
513	16.54%	88.70%	29.33%	78.94%	0.05	0.53	01.05
520	15.79%	88.70%	28.38%	78.79%	0.04	0.53	01.04

525	15.79%	88.91%	28.77%	78.83%	0.05	0.53	01.05
**Puncture-to-end time (PET)**							
35	92.48%	23.45%	25.52%	91.67%	0.16	0.62	1.16
37	90.23%	26.23%	25.75%	90.44%	0.17	0.62	1.16
38	89.47%	28.78%	26.27%	90.60%	0.18	0.62	1.18
39	87.97%	29.85%	26.23%	89.74%	0.18	0.62	1.18
40	87.22%	30.28%	26.19%	89.31%	0.18	0.62	1.17
45	82.71%	33.26%	26%	87.15%	0.16	0.62	1.16
46	82.71%	34.33%	26.32%	87.50%	0.17	0.62	1.17
47	81.95%	35.39%	26.46%	87.37%	0.17	0.62	1.17
48	80.45%	36.25%	26.35%	86.73%	0.17	0.62	1.17
49	80.45%	37.10%	26.62%	87%	0.18	0.62	1.18
50	79.70%	37.95%	26.70%	86.83%	0.18	0.62	1.18
51	79.70%	38.81%	26.97%	87.08%	0.19	0.62	1.19
52	78.95%	39.45%	26.99%	86.85%	0.18	0.62	1.18
53	51.13%	68.87%	31.78%	83.25%	0.20	0.62	1.20
54	51.13%	69.51%	32.23%	83.38%	0.21	0.62	1.21
55	49.62%	70.36%	32.20%	83.12%	0.20	0.62	1.20
56	48.12%	70.58%	31.68%	82.75%	0.19	0.62	1.19
57	45.86%	71.43%	31.28%	82.31%	0.17	0.62	1.17
58	45.11%	72.49%	31.75%	82.32%	0.18	0.62	1.18
59	44.36%	72.71%	31.55%	82.17%	0.17	0.62	1.17
60	42.86%	72.92%	30.98%	81.82%	0.16	0.62	1.16
